# Cost-utility and budget impact analyses of significant fibrosis detection in individuals with metabolic syndrome or obesity in Thailand

**DOI:** 10.1371/journal.pone.0344985

**Published:** 2026-03-23

**Authors:** Chayanis Kositamongkol, Pichaya Tantiyavarong, Alissa Ratanatawan, Pimsiri Sripongpun, Prawej Mahawithitwong, Prawat Kositamongkol, Surasak Saokaew, Pochamana Phisalprapa

**Affiliations:** 1 Department of Clinical Epidemiology, Faculty of Medicine, Thammasat University, Pathumthani, Thailand; 2 Division of Ambulatory Medicine, Department of Medicine, Faculty of Medicine Siriraj Hospital, Mahidol University, Bangkok, Thailand; 3 Department of Community Medicine and Family Medicine, Faculty of Medicine, Thammasat University, Pathumthani, Thailand; 4 Gastroenterology and Hepatology Unit, Division of Internal Medicine, Faculty of Medicine, Prince of Songkla University, Songkhla, Thailand; 5 Hepatopancreatobiliary and Transplant Surgery Unit, Division of General Surgery, Department of Surgery, Faculty of Medicine Siriraj Hospital, Mahidol University, Bangkok, Thailand; 6 Division of Social and Administrative Pharmacy (SAP), Department of Pharmaceutical Care, School of Pharmaceutical Sciences, University of Phayao, Phayao, Thailand; 7 Center of Excellence in Bioactive Resources for Innovative Clinical Applications, Chulalongkorn University, Bangkok, Thailand; 8 Unit of Excellence on Clinical Outcomes Research and IntegratioN (UNICORN), School of Pharmaceutical Sciences, University of Phayao, Phayao, Thailand; Kaohsiung Medical University Hospital, TAIWAN

## Abstract

**Introduction:**

Evidence on screening for significant fibrosis in individuals with metabolic syndrome or obesity at risk of metabolic dysfunction-associated steatotic liver disease is limited in low- and middle-income countries. We conducted a cost-utility analysis and a 5-year budget impact analysis of 3 one-time screening strategies versus no screening in Thai adults with metabolic syndrome or obesity.

**Methods:**

We built a lifetime economic model from a societal perspective to estimate quality-adjusted life years (QALYs) and costs. Strategies were: (1) Fibrosis-4 index (FIB-4) followed by transient elastography (TE), (2) Steatosis-Associated Fibrosis Estimator score followed by TE, and (3) TE alone. Inputs came from a literature review and primary data analysis. Costs and outcomes were discounted at 3% annually. Incremental cost-effectiveness ratios (ICERs) were compared with a willingness-to-pay threshold of 160,000 THB (4,619 USD) per QALY gained. One-way and probabilistic sensitivity analyses were undertaken, and a 5-year budget impact analysis was performed from the payer perspective.

**Results:**

In metabolic syndrome, FIB-4 + TE yielded an ICER of 104,588 THB (3,019 USD) per QALY gained versus no screening. The Steatosis-Associated Fibrosis Estimator score plus TE yielded 128,274 THB (3,703 USD). Extended dominance identified FIB-4 + TE as the sole cost-effective strategy. In obesity, all strategies were cost-effective, with TE alone preferred. The transition from fibrosis stage F3 to F4 most influenced ICERs. The probability that FIB-4 + TE was cost-effective ranged from 59% to 78%. Estimated annual budget impact over 5 years ranged from 564 to 2,314 million THB (16.3–66.8 million USD).

**Conclusions:**

One-time screening was not uniformly cost-effective. In metabolic syndrome, only FIB-4 + TE was cost-effective. In obesity, all strategies were cost-effective. Given non-robustness in the estimated cost-effectiveness and the substantial budget impact, implementation should balance expected health gains against affordability.

## Introduction

Metabolic syndrome (MetS) and obesity pose rising global public health challenges that substantially increase risk of metabolic dysfunction-associated steatotic liver disease (MASLD) and its progression to liver fibrosis [1]. Metabolic dysfunction drives MASLD pathogenesis and progression [2]. A recent guideline recommended screening for MASLD and liver fibrosis in individuals with cardiometabolic risk factors such as overweight or obesity, dysglycemia or type 2 diabetes, hypertriglyceridemia, low high-density lipoprotein cholesterol, and hypertension [3]. Early detection of fibrosis is critical because fibrosis stage predicts adverse outcomes, including hepatocellular carcinoma (HCC) and liver-related mortality. Effective screening and early identification of significant fibrosis (stage ≥ 2) enable timely interventions to reduce cancer incidence and other MASLD-related complications.

Moderate to advanced fibrosis has well-established prognostic significance: mortality rate ratios progressively increase from 2.52 (95% CI, 1.85–3.42) at stage F2, to 3.48 (95% CI, 2.51–4.38) at F3, and 6.40 (95% CI, 4.11–9.95) at F4 [4]. Despite its diagnostic accuracy, liver biopsy remains impractical for routine screening because of its invasiveness, cost, and limited feasibility [3]. As a result, noninvasive tests, particularly transient elastography (TE), have gained prominence for fibrosis evaluation due to their safety and accessibility. TE also quantifies hepatic steatosis through its controlled attenuation parameter and assesses fibrosis severity via liver stiffness measurement. Multiple noninvasive scoring systems—Fibrosis-4 (FIB-4) index, Aspartate Aminotransferase to Platelet Ratio index, Nonalcoholic Fatty Liver Disease Fibrosis Score, LiverRisk, Steatosis-Associated Fibrosis Estimator (SAFE) score, and Metabolic Dysfunction-Associated Fibrosis 5 score—are recommended for fibrosis risk stratification in clinical practice. Among these, we selected FIB-4 and SAFE as the primary strategies because their strengths are complementary. FIB-4 is a simple, widely validated score derived from routinely available laboratory tests and is recommended in multiple guidelines [3,5]. SAFE, a newer risk estimator, shows higher sensitivity for detecting fibrosis stage ≥ 2 and for predicting liver-related and cardiovascular outcomes, outperforming FIB-4 in recent validation studies [6].

The global burden of MASLD continues to rise, especially in low- and middle-income countries, where it imposes substantial health and economic challenges [7,8]. MASLD contributes significantly to disability-adjusted life years and accounts for around 44% of liver-related mortality worldwide [7]. Cardiovascular disease and nonhepatic cancers are the leading causes of death in patients with MASLD [2]. This epidemiological trend underscores the need for refined health policies and targeted prevention strategies in resource-constrained settings. Although studies in high-income countries have examined the cost-effectiveness of MASLD screening and fibrosis detection [9,10], generalizing these findings to resource-limited health systems is uncertain. Additionally, despite prior evidence demonstrating ultrasonography’s cost-effectiveness for MASLD screening in Thailand [11], systematic screening for MASLD and liver fibrosis is not implemented within the Thai healthcare system. Furthermore, the cost-effectiveness of detecting significant fibrosis among individuals with MetS or obesity within Thailand’s healthcare system remains unclear.

We therefore focused our economic evaluation on screening strategies for significant fibrosis in individuals with MetS or obesity in Thailand. While prior studies have assessed noninvasive tests for fibrosis in MASLD populations [12,13], few economic evaluations address these high-risk subgroups in the Thai context. This study aimed to perform a comprehensive cost-utility analysis and budget impact assessment of TE–based screening for significant fibrosis in these groups. Our findings are intended to inform evidence-based policy decisions and optimize resource allocation for MASLD and fibrosis management in resource-limited settings.

## Materials and methods

This study followed the Consolidated Health Economic Evaluation Reporting Standards 2022 Statement (**S1 File**) [14]. It was approved by the Ethics Committee of the Faculty of Medicine Siriraj Hospital, Mahidol University (Si 990/2023) and by the Thammasat University Human Research Ethics Committee (124/2024). The requirement for informed consent was waived due to the retrospective nature of the study and the use of de-identified participant data. The data was accessed for research purposes between June 1, 2024 and December 31, 2024.

### Overall description

**Fig 1:**
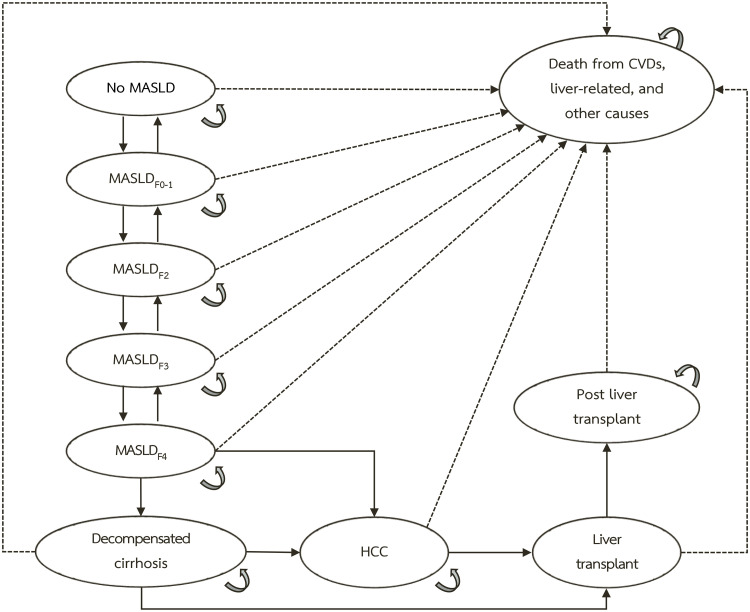
A Markov model. Ovals represent the MASLD-related health states. Solid arrows indicate the possible transitions that individuals may undergo within the model, while dashed arrows depict the probabilities of transitioning to the death state. **Abbreviations:** CVD, cardiovascular disease; F, fibrosis stage; HCC, hepatocellular carcinoma; MASLD, metabolic dysfunction-associated steatotic liver disease.

We conducted a cost-utility analysis comparing screening strategies for significant fibrosis versus no screening in individuals with MetS or obesity. A decision tree and a Markov model (**Fig 1** and **S2 File**) simulated patient trajectories over a lifetime horizon from a societal perspective, as per Thai Health Technology Assessment guidelines [15]. Key outcomes were life expectancy, total lifetime costs, and total lifetime quality-adjusted life years (QALYs). We calculated incremental cost-effectiveness ratios (ICERs) in 2023 Thai baht and United States dollars (USD) per QALY gained. We examined extended dominance by first ranking all strategies according to their effectiveness measured in QALYs and their costs. Any strategy that was more costly and less effective than another was excluded due to simple dominance. Next, we calculated ICERs by comparing each remaining strategy to the next less costly alternative. Extended dominance occurs when a strategy has an ICER higher than that of a more effective strategy, indicating it is less efficient and should be excluded. Only strategies with ICERs below the willingness-to-pay threshold were considered cost-effective. For this analysis, we used a threshold of 160,000 THB (4,619 USD) per QALY gained [15], based on an exchange rate of 34.64 THB per 1 USD.

Additionally, using the decision tree, we calculated the cost per true positive case detected to further inform policymakers regarding program planning. All analyses were performed in Microsoft Excel 365.

### Screening strategies for significant fibrosis detection

We evaluated 3 one‐time screening strategies for detecting fibrosis stage ≥ 2:

1. FIB-4 index followed by TE (“FIB-4 + TE”).
2. SAFE score followed by TE (“SAFE + TE”).
3. TE alone (“TE alone”).

The FIB-4 index is calculated from age, aspartate aminotransferase (AST), alanine aminotransferase (ALT), and platelet count [3,16]. The SAFE score incorporates 3 additional variables—body mass index, history of diabetes, and serum globulin—into the formula [17]. The thresholds for detecting fibrosis stage ≥ 2 used in this study were FIB-4 ≥ 1.3 [3] SAFE ≥ 0 [6,17], and a liver stiffness measurement ≥ 7.0 kPa on TE [18–20].

In base‐case analyses, 90% of the cohort underwent FIB-4 or SAFE screening, and 90% of those meeting the respective cutoff proceeded to TE [3,6]. The TE‐alone strategy assumed an 80% screening rate. We varied all screening rates to assess their impact on cost‐effectiveness.

### Target population of screening policy

The base-case population comprised individuals with MetS defined by National Cholesterol Education Program Adult Treatment Panel III criteria [21,22]. Scenario analyses included individuals with obesity, defined as body mass index ≥ 25 kg/m². We set the base-case screening age at 50 years, informed by primary cross-sectional data collected from patients attending Siriraj Hospital. In MetS, adults aged 50–59 years had the highest prevalence of MASLD with fibrosis stage ≥ 2 detected by TE. This age also allows time for lifestyle interventions to confer benefit. Accordingly, the base-case screening age for individuals with obesity was set at 50 years. Scenario analyses varied the screening age in 10-year increments from 30 to 80 years.

### Economic models

A decision tree linked to a Markov model estimated lifetime costs and outcomes for 3 screening strategies versus no screening (**Fig 1** and **S2 File**). Individuals were classified by screening result—positive or negative—using each test’s sensitivity and specificity to account for false positives and false negatives [18,23]. All individuals with a positive initial screen (FIB-4 ≥ 1.3 or SAFE ≥ 0)—including both true positives and false positives—were referred to TE according to the strategy-specific screening rates in the model. Those with a liver stiffness measurement ≥ 7.0 kPa on TE and true fibrosis stage F2–F4 received the treatment intervention. Individuals with liver stiffness measurement ≥ 7.0 kPa due to TE false positives incurred intervention costs without benefit. All others—comprising individuals with true negative results, false negative results, or a liver stiffness measurement < 7.0 kPa—followed the natural history of MASLD without intervention.

The Markov model (**Fig 1**) simulated long-term MASLD progression across 10 health states, adapted from prior Thai studies [8,11,24]. States ranged from no MASLD through fibrosis stages 0–4, decompensated cirrhosis, HCC, liver transplant, post-transplant, and death from cardiovascular disease, liver-related causes, or other causes. Patients could remain in their current state, progress to more advanced states, or regress to less advanced states until death, as specified in **Fig 1**. The model used 1-year cycles over a lifetime horizon, with costs and outcomes discounted at 3% annually. The Markov model accumulated total lifetime costs and total lifetime QALYs for each strategy.

### Model input parameters

Table 1 lists input parameters, including screening test performance (sensitivity and specificity), epidemiological data, treatment effectiveness, transition probabilities, utility values, and costs. These parameters were sourced from published literature and primary data collected at Siriraj Hospital in Bangkok [36].

**Table 1:** Model’s input parameters.

Input parameter	Distribution	Base-case value	SE or range	Reference
*Test performance* ^*a*^				
FIB-4 index ≥ 1.3 Sensitivity Specificity	Beta Beta	66.0% 65.0%	1.2% 1.4%	[23]
SAFE score ≥ 0 Sensitivity Specificity	Beta Beta	87.3% 35.1%	0.8% 1.4%	[23]
TE, LSM ≥ 7 kPa Sensitivity Specificity	Beta Beta	80.1% 73.0%	2.1% 2.6%	[18]
*Screening rate*				
FIB-4 or SAFE	Beta	90%	50–100%	Assumption
Follow-up TE among those with FIB-4 ≥ 1.3 or SAFE ≥ 0	Beta	90%	65–100%	Assumption
TE alone	Beta	80%	30–100%	Assumption
*Epidemiological data*				
Prevalence				
MASLD age 18–39.9 years age 40–59.9 years age ≥ 60 years	Beta Beta Beta	0.353 0.348 0.244	0.013 0.002 0.004	[8]
MASLD with significant fibrosis MetS age 30–39.9 years age 40–49.9 years age 50–59.9 years age 60–69.9 years age 70–79.9 years age ≥ 80 years Obesity age 30–39.9 years age 40–49.9 years age 50–59.9 years age 60–69.9 years age 70–79.9 years age ≥ 80 years	Beta Beta Beta Beta Beta Beta Beta Beta Beta Beta Beta Beta	0.174 0.108 0.116 0.114 0.105 0.407 0.250 0.227 0.241 0.361 0.324 0.522	0.079 0.038 0.020 0.020 0.029 0.095 0.056 0.043 0.029 0.028 0.044 0.104	Primary data,S3 File
Incidence of MASLD(cases per 1,000 patient-years) MetS Obesity	Beta Beta	54.9 50.9	16.9 3.3	[11,25] [26]
*Treatment effectiveness*				
RRR of lifestyle modification	Log-normal	0.204	0.124	[27], S4 File
Transition probability				
MetS: No MASLD → MASLD_F0_ Obesity: No MASLD → MASLD_F0_	Beta Beta	0.055 0.051	0.017 0.003	[11,25] [26]
MASLD_F0_ → No MASLD MASLD_F0_ → MASLD_F1_	Beta Beta	0.024 0.063	0.013 0.025	[28] [28]
MASLD_F1_ → MASLD_F0_ MASLD_F1_ → MASLD_F2_	Beta Beta	0.025 0.067	0.017 0.025	[28] [28]
MASLD_F2_ → MASLD_F1_ MASLD_F2_ → MASLD_F3_	Beta Beta	0.045 0.056	0.022 0.024	[28] [28]
MASLD_F3_ → MASLD_F2_ MASLD_F3_ → MASLD_F4_/CC	Beta Beta	0.058 0.044	0.024 0.021	[28] [28]
MASLD_F4_/CC → MASLD_F3_ MASLD_F4_/CC → DC MASLD_F4_/CC → HCC	Beta Beta Beta	0.046 0.043 0.008	0.022 0.041 0.017	[28] [12] [12]
DC → HCC DC → LT (age ≤ 70 years)	Beta Beta	0.029 0.003	0.045 0.012	[12] [12]
HCC → LT (age ≤ 70 years)	Beta	0.012	0.027	[12]
Utilities				
No MASLD: MetS No MASLD: Obesity	Beta Beta	0.890 0.908	0.003 0.004	[11,29] [30]
MetS with MASLD_F0-F3_	Beta	0.840	0.071	[11,31]
Obesity with MASLD_F0-F3_	Beta	0.908	0.131	[30]
MASLD_F4_/CC	Beta	0.748	0.042	[24]
DC	Beta	0.603	0.022	[32]
HCC	Beta	0.380	0.015	[33]
LT	Beta	0.570	0.015	[33]
Post LT	Beta	0.683	0.015	[32]
Costs				
*Direct medical costs, 2023 THB (USD)*				
Screening tests FIB-4 index SAFE score Laboratory test AST ALT Globulin Platelet count Transient elastography	Gamma Gamma Gamma Gamma Gamma	271.0 (7.8) 355.4 (10.3) 84.4 (2.4) 84.4 (2.4) 84.4 (2.4) 102.1 (2.9) 2,000.0 (57.7)	NA NA 21.1 (0.6) 21.1 (0.6) 21.1 (0.6) 25.5 (0.7) 500.0 (14.4)	Calculation Calculation [34] [34] [34] [34] Primary data
Lifestyle modification program, THB (USD) per year	Gamma	1,426.8 (41.2)	356.7 (10.3)	[34]
Treatment costs per year MetS Obesity MetS with MASLD_F0-F3_ Obesity with MASLD_F0-F3_ MetS with MASLD_F4_ Obesity with MASLD_F4_ DC HCC LT Post LT	Gamma Gamma Gamma Gamma Gamma Gamma Gamma Gamma Gamma Gamma	2,668.5 (77.0) 0 8,294.1 (239.4) 14,583.7 (421.0) 38,394.5 (1,108.4) 37,568.2 (1,084.6) 151,164.1 (4,363.9) 184,822.2 (5,335.6) 683,432.3 (19,729.9) 110,287.1 (3,183.9)	667.1 (19.3) 0 2,073.5 (59.9) 3,645.9 (105.3) 9,598.6 (277.1) 9,392.0 (271.1) 37,791.0 (1,091.0) 46,205.5 (1,333.9) 170,858.1 (4,932.5) 27,571.8 (796.0)	Primary data Assumption Primary data Primary data Primary data Primary data [24,35] [24,35] [24,35] [24,35]
*Direct non-medical costs, 2023 THB (USD) per visit*				
Secondary hospital Food Transportation Tertiary hospital Food Transportation	Gamma Gamma Gamma Gamma	33.1 (1.0) 91.2 (2.6) 66.2 (1.9) 179.7 (5.2)	4.0 (0.1) 5.2 (0.1) 6.7 (0.2) 14.6 (0.4)	[34] [34] [34] [34]

^a^The sensitivity and specificity of the tests were compared to liver biopsy, a reference standard for liver fibrosis diagnosis.

**Abbreviations:** ALT, alanine aminotransferase; AST, aspartate aminotransferase; DC, decompensated cirrhosis; F, fibrosis stage; FIB-4, fibrosis-4 index; HCC, hepatocellular carcinoma; LSM, liver stiffness measurement; LT, liver transplantation; MASLD, metabolic dysfunction-associated steatotic liver disease; MetS, metabolic syndrome; RRR, relative risk reduction; NA, not applicable; SAFE, steatosis-associated fibrosis estimator score; SE, standard error; TE, transient elastography; THB, Thai baht; USD, United States dollars

#### Screening test performance: sensitivity and specificity.

Sensitivity and specificity for FIB-4 and SAFE were obtained from a multicenter study by Alkhouri et al. [23]. Diagnostic accuracy of TE was derived from a meta-analysis by Selvaraj et al. [18], using liver biopsy as the gold standard.

#### Epidemiological data, natural course of MASLD, and mortality.

Age‐specific MASLD prevalence was obtained from prior Thai economic evaluations [8,11]. Incidence among individuals with MetS and obesity was sourced from Hamaguchi et al. [25] and Li et al. [26], respectively. The significant fibrosis detection rate was calculated from primary de-identified data on 743 individuals with MetS and 799 individuals with obesity at Siriraj Hospital between January 1, 2018 and October 31, 2023 (**S3 File**). Natural history parameters came from recent systematic reviews and meta‐analyses [12,28]. We assumed liver transplantation was restricted to patients under 70 years of age [32]. Baseline mortality rates were derived from World Health Organization life tables and adjusted for MetS– and obesity‐related mortality using data from previous studies [37,38]. In patients with MASLD, mortality was estimated by applying hazard ratios of 1.29 (95% CI, 1.04–1.59) for non‐cirrhotic disease and 3.13 (95% CI, 1.08–9.12) for cirrhotic disease [39]. Cause‐specific mortality distributions for cardiovascular and liver‐related deaths were based on Tampi et al. [40] and Huang et al. [41]. Post‐transplant mortality rates were obtained from Thai patient cohorts [42,43]. All rates were converted to annual transition probabilities for the Markov model.

#### Treatment effectiveness.

In the base-case analysis, all individuals screening positive (FIB-4 ≥ 1.3, SAFE ≥ 0, or liver stiffness measurement ≥ 7.0 kPa) received a lifestyle intervention targeting weight loss. Relative risk reductions in fibrosis progression were derived from Vilar-Gomez et al., which correlated the degree of weight loss with histological improvement [27]. Detailed calculation is provided in **S4 File**. The model applied these effects by multiplying the transition probabilities for progression from fibrosis stage 2–3, 3–4, and 4 to decompensated cirrhosis by one minus the relative risk reduction in those with true fibrosis stage 2–4. Treatment effectiveness was assumed uniform across fibrosis stages and applied only among those who adhere to the treatment. Uncertainty in treatment adherence was explored through one-way sensitivity analysis by varying the rate from 60% to 100%.

#### Utilities.

Utility scores were sourced from published studies. For MetS, we adopted values from the Korean Health and Nutrition Examination Survey using the EuroQol questionnaire [29]. Utility scores for obesity came from a representative Thai cohort study [30]. Utilities for cirrhosis and related hepatic complications were obtained from observational studies and a Thai economic evaluation [24,32,33].

#### Costs.

From a societal perspective, we included direct medical and nonmedical costs. Direct medical costs were sourced from primary data analyses and published local studies. Direct nonmedical costs, including food and transportation, were taken from the Standard Cost Lists for Health Technology Assessment in Thailand [34]. All costs were adjusted to 2023 values using the consumer price index [44]. We assumed individuals with obesity without MASLD incurred no healthcare costs. Management costs for MetS and for care after MASLD diagnosis were estimated from primary analyses of 780 patients with MetS or obesity and 91 patients with MASLD_F4_ in the Siriraj Hospital database (January 1, 2018–October 31, 2023; **S5 File**). We calculated these costs by extracting disease-management charges from the database and converting charges to costs using a cost-to-charge ratio of 1. We adopted a ratio of 1 because cost-to-charge ratios specific to Thai university hospitals are limited, and existing literature indicates that this ratio for Thai hospitals varies from approximately 0.6 to 1.2, depending on service category, hospital size, and level of care [45,46]. Costs for decompensated cirrhosis, HCC, and liver transplantation were obtained from local literature [24,35].

We calculated the costs of FIB-4 and SAFE from unit costs of required laboratory tests—AST, ALT, and platelet count for FIB-4; plus globulin for SAFE. The total per-screening costs were 271.0 THB (7.8 USD) for FIB-4 and 355.4 THB (10.3 USD) for SAFE [34]. We estimated the cost of TE at 2,000 THB (57.7 USD). All input parameters are detailed in Table 1.

### Model validation

The decision tree and Markov model underwent face, internal, and external validity assessments. Experts reviewed the framework, model structures, assumptions, inputs, algorithms, and outputs. Model outcomes were compared with published real‐world data to ensure accuracy. The model validation process is shown in **S6 File**.

### Sensitivity analyses

We performed one-way deterministic sensitivity analyses, varying each parameter across its 95% confidence interval; treatment cost parameters were varied by ± 25% to assess effects on ICERs. Results were summarized with tornado diagrams. The model’s robustness was further evaluated by varying the adherence rate of lifestyle intervention across range of 60% to 100% and cost-to-charge ratio which applied to the costs derived from the Siriraj Hospital database across range of 0.6 to 1.2.

A probabilistic sensitivity analysis used 1,000 Monte Carlo simulations: beta distributions for test accuracy and transition probabilities, log‐normal for intervention effectiveness, and gamma for cost data, following Thai Health Technology Assessment guidelines [15]. Results are presented as cost-effectiveness planes. To identify the optimal option among the three competing screening strategies, we calculated the net monetary benefit for each strategy versus no screening across willingness-to-pay thresholds from 0 to 300,000 THB (8,661 USD), using incremental costs and QALYs from the simulations. The net monetary benefit was calculated as: net monetary benefit = (willingness-to-pay threshold * QALY gained) – incremental cost. The probability that each strategy is optimal—defined as the highest net monetary benefit at a given threshold—is displayed using cost-effectiveness acceptability curves. This approach incorporates parameter uncertainty into the interpretation.

### Threshold analysis

For strategies with ICERs exceeding the Thailand’s current willingness‐to‐pay threshold, we conducted threshold analyses to identify the maximum screening cost at which each strategy remains cost‐effective.

### Budget impact analysis

We conducted a 5-year budget impact analysis from the payer perspective. The analysis estimated the additional budget required to implement screening strategies by counting only direct medical costs of screening for fibrosis stage ≥ 2 in individuals with MetS or obesity. We excluded costs of post-screening management, disease treatment, and direct non-medical items. We estimated the target population from the total Thai population [47], adjusted for disease prevalence and incidence. An open-cohort model projected the screened population over 5 years. In year 1, we included the prevalent population with MetS, obesity, or both [48–50]; in years 2–5, we added new eligible individuals based on incidence rates [48,51]. The target population size estimation is described in **S7 File**. We varied uptake of initial TE screening from 30% to 100%. Uptake of clinical scoring tests (FIB-4 or SAFE) ranged from 50% to 100%, and follow-up TE after FIB-4 ≥ 1.3 or SAFE ≥ 0 ranged from 65% to 100%. We did not apply discounting because discounted costs would not reflect the actual budget impact within a given year [52]. Outcomes are reported as the average annual budget and the total 5-year budget, in THB and USD.

## Results

### Cost-utility analysis

In MetS, SAFE + TE yielded the highest QALYs, but TE alone incurred the highest lifetime costs. No screening had the lowest QALYs and lowest costs. FIB-4 + TE and SAFE + TE were cost-effective versus no screening at Thailand’s current willingness-to-pay threshold of 160,000 THB (4,619 USD) per QALY gained. Their ICERs were 104,588 THB (3,019 USD) and 128,274 THB (3,703 USD) per QALY gained, respectively. Extended-dominance analysis showed SAFE + TE and TE alone were not cost-effective in MetS. In obesity, all 3 strategies were cost-effective, and TE alone provided the highest QALYs. Detailed results are presented in Table 2.

**Table 2:** Cost-utility analysis outcomes in individuals with metabolic syndrome and individuals with obesity at screening age of 50 years.

Outcome	Life expectancy (years)	Total lifetime cost^a^, THB (USD)	Total QALYs^a^	ICER^b^, THB (USD) per QALY gained	ICER^b^, THB (USD) per QALY gained
*Metabolic syndrome*					
No screening	26.81	109,897.3 (3,172.6)	15.224		
FIB-4 + TE	26.83	110,748.6 (3,197.2)	15.232	104,587.67^c^ (3,019.33)	104,587.67^c^ (3,019.33)
SAFE+TE	26.83	111,200.1 (3,210.2)	15.234	128,274.36^c^ (3,703.13)	223,899.61^d^ (6,463.73)
TE alone	26.83	112,183.4 (3,238.6)	15.233	255,221.17^c^ (7,367.94)	Dominated^e^
Obesity					
No screening	29.05	168,147.4 (4,854.2)	16.841		
FIB-4 + TE	29.07	168,700.9 (4,870.2)	16.853	46,413.47^c^ (1,339.90)	46,413.47^c^ (1,339.90)
SAFE+TE	29.08	169,013.5 (4,879.2)	16.858	51,743.86^c^ (1,493.79)	64,955.01^d^ (1,875.18)
TE alone	29.09	169,492.4 (4,893.1)	16.862	65,728.93^c^ (1,897.52)	128,567.98^e^ (3,711.61)

^a^Discounted at 3% annually, the total lifetime costs and QALYs are rounded to 1 and 3 decimal places, respectively.

^b^ICERs were originally computed in Microsoft Excel 365 and are presented rounded to 2 decimal places.

^c^Compared to no screening

^d^Compared to FIB-4 + TE

^e^Compared to SAFE+TE

**Abbreviations:** FIB-4, fibrosis-4 index; ICER, incremental cost-effectiveness ratio; QALY, quality-adjusted life-year; SAFE, steatosis-associated fibrosis estimator score; TE, transient elastography; THB, Thai baht; USD, United States dollars

The cost per true positive case detected for each screening strategy showed that, despite having the lowest proportion of true positives among screened individuals, FIB-4 + TE produced the lowest cost for both MetS and obesity individuals: 15,003.47 THB (433.13 USD) and 7,677.63 THB (221.64 USD) per true positive case detected, respectively. This was followed by the TE alone strategy and the SAFE + TE strategy (**S8 File**).

### Scenario analyses

**Fig 2:**
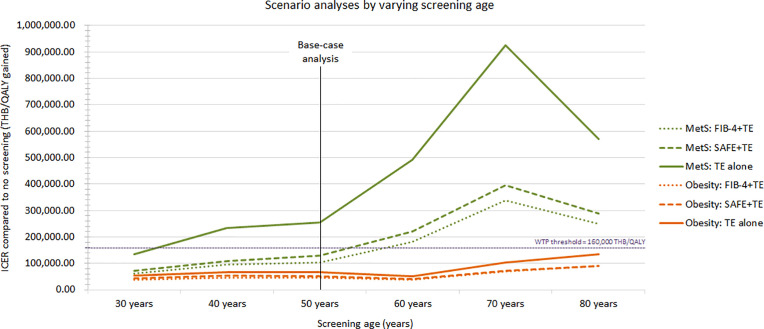
Scenario analyses. The graph showing ICERs of screening strategies compared to no screening across screening ages ranging from 30 to 80 years.

Cost‐effectiveness of fibrosis screening was greatest at younger ages. At age 30, all 3 strategies met cost‐effectiveness criteria. In MetS, TE alone ceased to be cost‐effective at age 40, and both FIB-4 + TE and SAFE + TE lost cost‐effectiveness at age 60 and older. In obesity, all strategies stayed cost‐effective across ages 30–80 (**Fig 2** and **S8 File**).

Lower screening rates reduced lifetime costs and QALYs for each strategy, while higher rates increased both outcomes. However, the rank order of cost‐effectiveness among strategies was generally stable across the range of screening rates examined.

### Model validation

Experts in gastroenterology, hepatology, and health economics reviewed and validated the analysis framework, model structure, assumptions, inputs, and outputs. Algorithmic accuracy in Microsoft Excel 365 was confirmed by extreme value testing and stepwise tracing [15].

For external validation, the model projected MASLD incidence of 58.5 and 54.1 cases per 1,000 person‐years among individuals with MetS and obesity, respectively. These rates align with the 50.9 cases per 1,000 person‐years (95% CI, 44.8–57.4) reported by Li et al. [26] The model also predicted an annual HCC incidence of 1.08% to 1.12%, consistent with published rates of 0.50% to 2.26% in patients with metabolic dysfunction-associated steatohepatitis cirrhosis [53].

### Sensitivity analyses

#### One-way sensitivity analysis.

**Fig 3:**
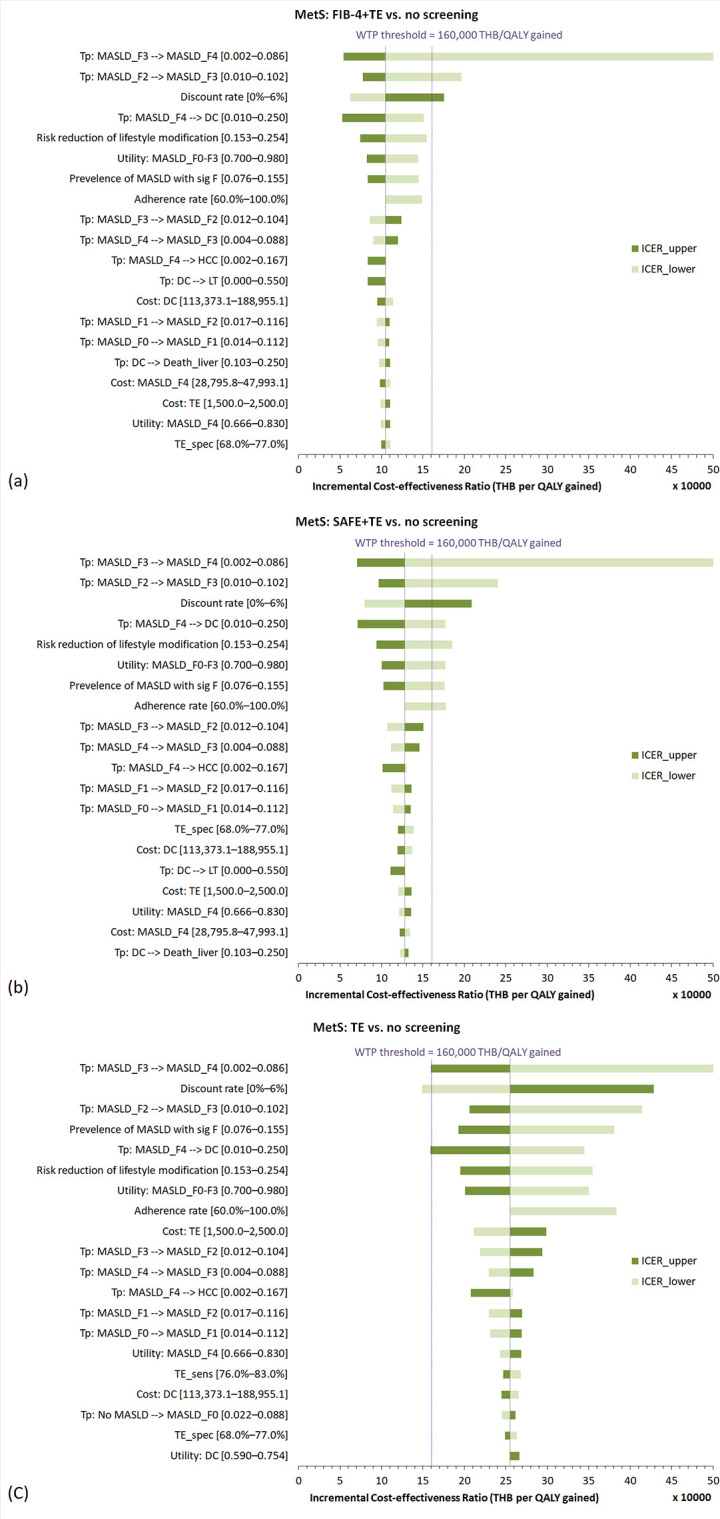
Tornado diagrams of (a) FIB-4 + TE, (b) SAFE+TE, and (c) TE alone compared to no screening, in individuals with metabolic syndrome. **Abbreviations:** DC, decompensated cirrhosis; F, fibrosis stage; FIB-4, fibrosis-4 index; LSM, liver stiffness measurement; MASLD, metabolic dysfunction-associated steatotic liver disease; MetS, metabolic syndrome; QALY, quality-adjusted life-year; SAFE, steatosis-associated fibrosis estimator score; sig F, significant fibrosis; spec, specificity; TE, transient elastography; THB, Thai baht; Tp, transition probability.

**Fig 4:**
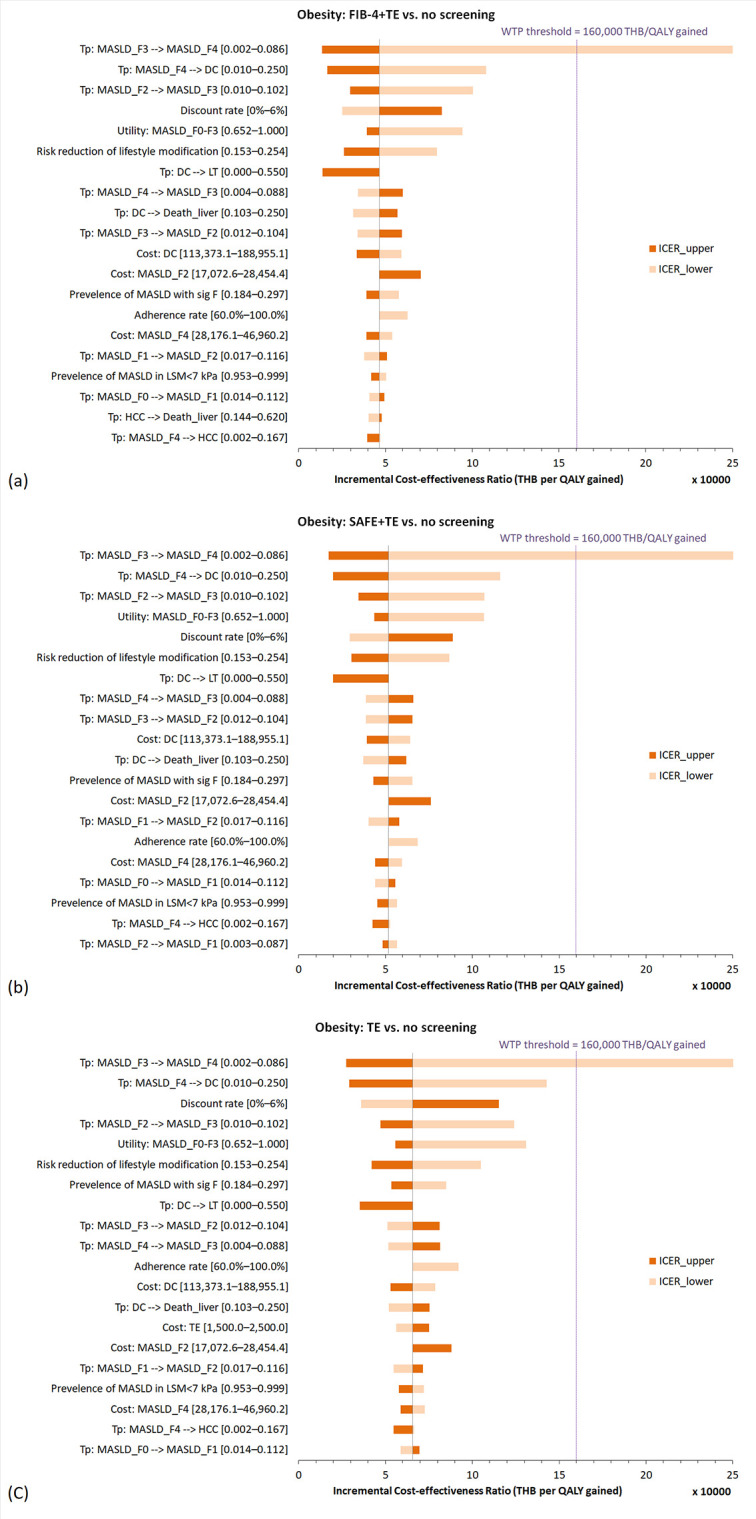
Tornado diagrams of (a) FIB-4 + TE, (b) SAFE+TE, and (c) TE alone compared to no screening, in individuals with obesity. **Abbreviations:** DC, decompensated cirrhosis; F, fibrosis stage; FIB-4, fibrosis-4 index; LSM, liver stiffness measurement; MASLD, metabolic dysfunction-associated steatotic liver disease; QALY, quality-adjusted life-year; SAFE, steatosis-associated fibrosis estimator score; sig F, significant fibrosis; spec, specificity; TE, transient elastography; THB, Thai baht; Tp, transition probability.

One‐way deterministic sensitivity analysis revealed that variations in fibrosis progression rates (MASLD_F3_ to MASLD_F4_, MASLD_F2_ to MASLD_F3_, and MASLD_F4_ to decompensated cirrhosis) and discount rate had the greatest impact on ICERs compared with no screening. Tornado diagrams are shown in **Fig 3** and **4**.

A decrease in the treatment adherence rate resulted in a corresponding increase in the ICERs for all screening strategies, suggesting that lower treatment adherence reduces the cost-effectiveness of screening interventions. In MetS, the ICERs of TE alone remained above the willingness-to-pay threshold across the entire varied range of adherence rates. Conversely, FIB-4 + TE remained consistently cost-effective, while SAFE + TE ceased to be cost-effective when the adherence rate dropped below 69.9%. In obesity, all screening strategies remained cost-effective regardless of the adherence rate variation. The results are summarized in **S9 File**.

In addition, when we varied the cost-to-charge ratio from 0.6 to 1.2, the results remained similar to those of the base-case analyses, in which a ratio of 1 was applied. Results are in **S9 File**.

#### Probabilistic sensitivity analysis.

We used 1,000 Monte Carlo simulations to evaluate uncertainty in cost-effectiveness outcomes. When compared with no screening, cost‐effectiveness planes showed that, in MetS, FIB-4 + TE was cost-effective in 59.0% of iterations, SAFE + TE in 48.2%, and TE alone in 11.4%. In obesity, these proportions were 77.9%, 76.1%, and 70.0%, respectively (**S10 File**).

**Fig 5:**
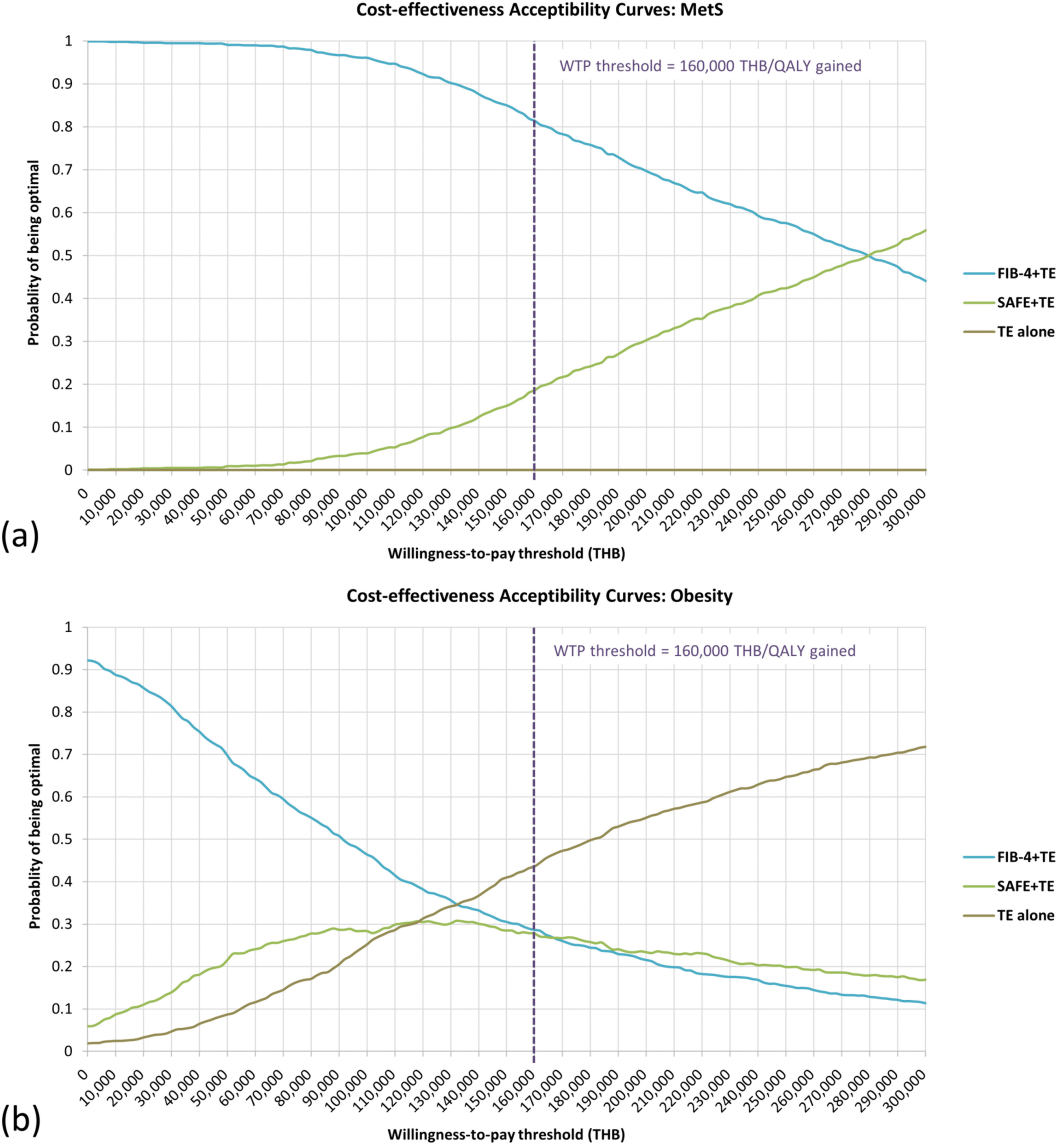
Cost-effectiveness acceptability curves. **(a)** Individuals with metabolic syndrome and (b) individuals with obesity. **Abbreviations:** FIB-4, fibrosis-4 index; MetS, metabolic syndrome; QALY, quality-adjusted life-year; SAFE, steatosis-associated fibrosis estimator score; TE, transient elastography; THB, Thai baht.

Cost‐effectiveness acceptability curves (**Fig 5**) showed the probability that each of the 3 screening strategies (excluding no screening) was optimal across willingness‐to‐pay thresholds from 0 to 300,000 THB (8,661 USD). Optimality was defined as the strategy achieving the highest net monetary benefit at each willingness-to-pay threshold. At 160,000 THB (4,619 USD) per QALY gained in MetS, FIB-4 + TE had an 81.4% probability of being optimal, SAFE + TE 18.6%, and TE alone 0%. In obesity, a divergence was observed between the ICER rankings and probabilistic optimality. Despite its highest ICER in Table 2, TE alone had the highest probability of being optimal at 43.6%, followed by FIB-4 + TE (28.6%) and SAFE + TE (27.8%). This indicated that when accounting for parameter uncertainty and using net monetary benefit approach, TE alone was the strategy most likely to be optimal at the current willingness-to-pay threshold.

### Threshold analysis

Cost-utility analysis demonstrated that, compared with no screening, TE alone was not cost‐effective in individuals with MetS. Threshold analysis indicated that reducing the cost of TE by at least 54.4%, from 2,000 THB (57.7 USD) to approximately 912 THB (26.3 USD), would lower its ICER below the willingness‐to‐pay threshold and render TE alone economically viable. The ICERs across a range of TE unit costs are presented in **S11 File**.

### Budget impact analysis

We used Thai prevalence rates of 18.0% for MetS [48], 33.4% for obesity [49], and 15.2% for both conditions [49,50]. Annual incidence among adults aged 50–79 years was 8.0% for MetS [48], 0.9% for obesity [51], and 4.5% for both conditions (range, 0.9%–8.0%) [48,51]. Using these inputs, we estimated 5-year target populations of 4.2 million with MetS, 7.2 million with obesity, and 3.4 million with both conditions. At screening rates of 90% for FIB-4 and SAFE and 80% for TE alone, the average annual budget impact was 714.6–1,335.2 million THB (20.6–38.5 million USD) for MetS. For obesity, it was 1,414.1–2,313.7 million THB (40.8–66.8 million USD). For both conditions, it was 563.9–1,097.0 million THB (16.3–31.7 million USD).

**Fig 6:**
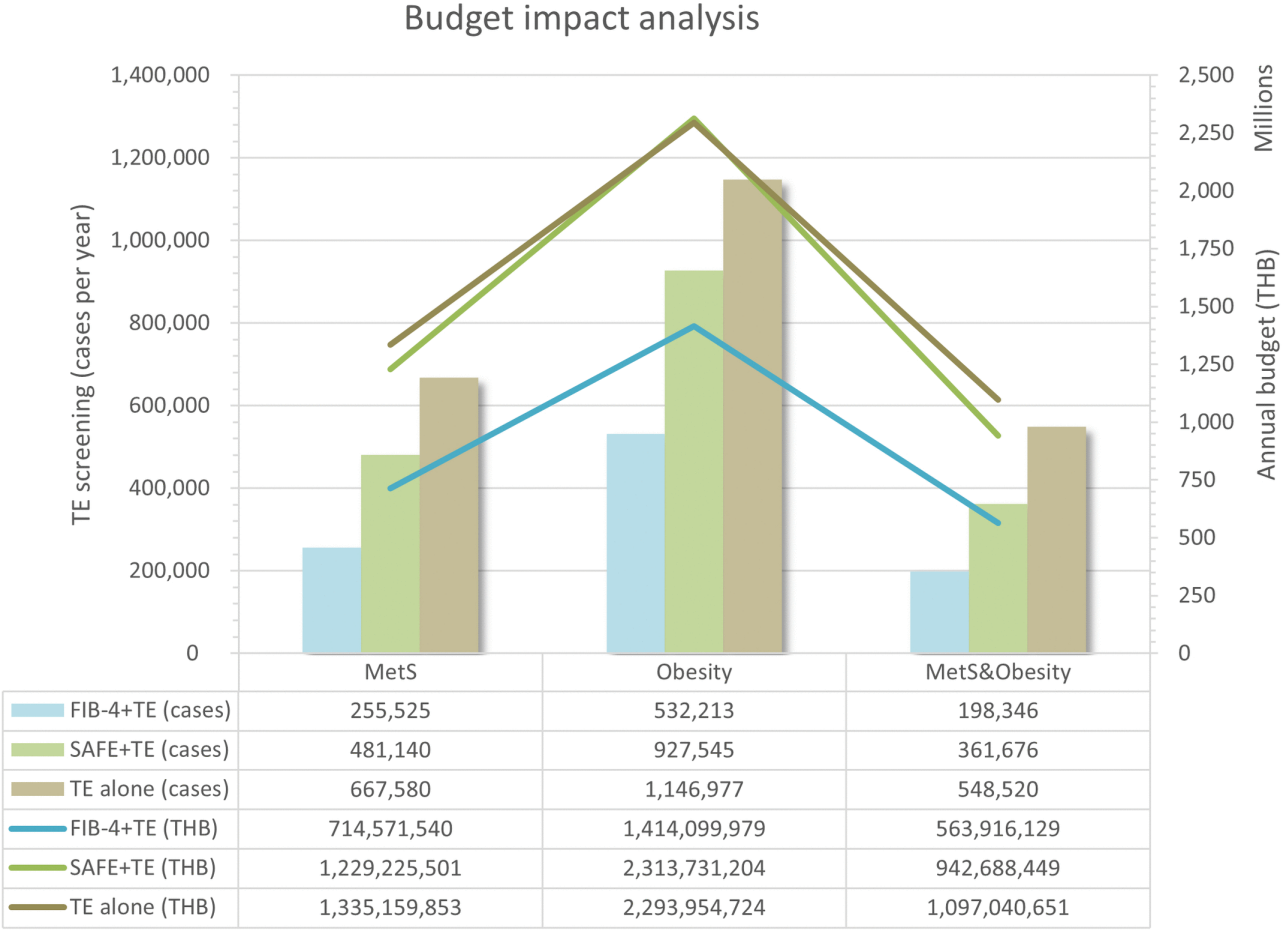
Budget impact analysis. Bar charts show the annual number of transient elastography screening cases for each strategy, and overlaid lines depict the corresponding annual budget impact of each strategy (in million THB). **Abbreviations:** FIB-4, fibrosis-4 index; MetS, metabolic syndrome; SAFE, steatosis-associated fibrosis estimator score; TE, transient elastography; THB, Thai baht.

FIB-4 + TE had the lowest budget impact, while SAFE + TE and TE alone had similar impacts (**Fig 6** and **S12 File**). At lower screening rates—50% for clinical scoring, 65% TE follow-up, and 30% for TE alone—the annual budget fell to 318.1–534.4 million THB (9.2–15.4 million USD) for MetS. For obesity, it was 621.3–999.1 million THB (17.9–28.8 million USD). For both conditions, it was 252.1–412.1 million THB (7.3–11.9 million USD). At ideal screening rates of 100%, the maximum annual budget was 1,669.0 million THB (48.2 million USD) for MetS. For obesity and for both conditions, the maxima were 2,867.4 million THB (82.8 million USD) and 1,371.3 million THB (39.6 million USD), respectively.

## Discussion

This study assessed the cost-effectiveness and budget impact of fibrosis screening in Thai MASLD populations at high risk—specifically, individuals with MetS or obesity. In MetS, compared to no screening, stepwise screening with FIB-4 or SAFE before TE was cost-effective; TE alone required more than a 50% cost reduction to meet the willingness-to-pay threshold. By contrast, in obesity, all 3 strategies—including FIB-4 + TE, SAFE + TE, and TE alone—were cost-effective without any reduction in TE cost. Incremental QALY gains from a single screening were modest, suggesting potential benefits of repeated assessments, although guidelines lack evidence to define optimal intervals [3,5]. These modest QALY gains contribute to non-robustness in cost-effectiveness, as reflected on the cost-effectiveness plane. In MetS, only 59% of probabilistic sensitivity analysis iterations found FIB-4 + TE to be cost-effective at the current willingness-to-pay threshold. Given the uncertainty in results, when interpreting cost-effectiveness using Thailand’s 2023 gross domestic product per capita of approximately 249,404 THB (7,200 USD) instead of the willingness-to-pay threshold of 160,000 THB (4,619 USD) per QALY gained, the probabilities of cost-effectiveness were increased from 11.4%–59.0% to 33.3%–82.9% for MetS and from 70.0%–77.9% to 83.2%–88.7% for obesity. Our findings underscore the value of tailored screening strategies that balance economic and clinical outcomes in resource-constrained settings.

Despite limited evidence in resource-constrained settings, our findings align with Decharatanachart et al. [54], who evaluated noninvasive tests for detecting advanced fibrosis and initiating HCC surveillance in MASLD populations. They reported that combined FIB-4 and TE yielded the lowest ICER and was cost-effective in Thailand for both the general MASLD population and those with obesity.

Consistent with previous cost-effectiveness studies [9,54], our analysis demonstrates that stepwise screening with FIB-4 followed by TE is the most cost-effective strategy in high-risk groups. Although TE alone provided higher QALYs, it incurred the greatest lifetime costs and was optimal only for individuals with obesity. This reflects TE’s higher sensitivity and specificity for fibrosis detection but greater expense, which may limit its cost-effectiveness unless reserved for those at highest risk [54].

Age at screening emerged as a critical determinant. Screening at age 30 years was cost-effective for all strategies. In MetS, TE alone lost cost-effectiveness from age 40, and both FIB-4 + TE and SAFE + TE were not cost-effective at age 60 and older. The cost-effectiveness of screening strategies tended to worsen (ICER increased) as screening age increased, despite rising MASLD and fibrosis prevalence with age [55]. This pattern reflects the increasing burden of comorbidities and competing mortality risks in older populations, which diminish the incremental benefit of early fibrosis detection and intervention. Notably, our primary data on significant fibrosis prevalence might be prone to minor selection bias due to limited sample sizes, potentially overestimating the prevalence in individuals aged ≥ 80 years. Although screening at younger ages was more cost‐effective, these benefits must be weighed against the higher incremental budget impact in resource‐constrained settings. This balanced approach informs public health investment by highlighting both economic efficiency and fiscal feasibility.

Our budget impact analysis highlights barriers to implementation. One-time screening for fibrosis in MetS demands about 1,000 million THB (30 million USD) per year and even more for obesity. Limited access to TE throughout Thailand further constrains feasibility. We estimated budget impact across wide screening uptake ranges (30% to 100%) to reflect real-world constraints, including device availability, limited trained personnel, and access barriers. In the future, if the policy is promoted with improved service accessibility and maximized device utilization at the national level, screening unit costs would likely decrease, enhancing cost-effectiveness. Unlike ultrasonography or other imaging techniques, TE can be operated and interpreted without radiologists. This characteristic enhances feasibility of national-scale screening given limited human resources. Additionally, restricting screening to individuals with obesity plus at least one additional metabolic risk factor could further reduce the overall budget impact. Our findings indicated that the annual budget decreased by approximately 40% to 48% when screening was limited to individuals with both MetS and obesity.

In Thailand, rising MASLD and obesity prevalence and constrained resources underscore the need for efficient allocation of screening tools. Our study confirmed that FIB-4 + TE had the highest probability of cost-effectiveness across both MetS and obesity cohorts. Given that TE devices are currently predominantly available in urban settings, the use of a simple, widely accessible scoring systems such as FIB-4 as a preliminary screening step is particularly suitable in the Thai context. Because laboratory tests required for the score calculation are broadly accessible even in rural areas, this approach facilitates early identification of individuals at risk and optimizes resource use.

Despite different analytical contexts, our findings on fibrosis screening cost‐effectiveness align with trends from high‐income countries [56,57]. Prior Thai research evaluated MASLD screening cost‐effectiveness in MetS populations [11]; our study instead targets fibrosis stage ≥ 2, a condition directly linked to adverse outcomes including increased mortality. Moreover, the earlier study assessed ultrasonography, whereas we selected TE for its superior accuracy in detecting fibrosis. Recent work [54] examined noninvasive tests for initiating surveillance in MASLD patients. Collectively, these studies emphasize for policymakers the importance of early fibrosis detection and timely intervention to halt progression and improve health‐related quality of life. However, most existing studies—including ours—focus on screening strategies and give limited attention to post-screening treatment pathways.

One-way sensitivity analysis identified post-screening intervention effectiveness as a key driver of screening cost-effectiveness. This underscores the need to evaluate diagnostic and therapeutic pathways when forming liver disease policy. The recent United States approval of resmetirom for significant and advanced fibrosis in metabolic dysfunction–associated steatohepatitis [56] postdates our analysis and was therefore not incorporated; it nonetheless offers promising new therapeutic options. Our model framework can be adapted to assess the cost‐effectiveness of adding resmetirom versus lifestyle modification alone, which is especially important in resource-limited settings.

Our study fills a critical gap by evaluating cost-effectiveness in populations with MetS and obesity, where few studies have examined screening for fibrosis in resource-limited settings. We used context-specific parameters for epidemiological data, health state utilities, and costs. Wherever possible, we derived parameters from studies conducted in the Thai population. For other inputs—such as MASLD incidence and utility values—we sourced estimates from closely comparable populations (primarily Asian) and had experts validate their relevance. Transition probabilities were drawn from the latest systematic reviews to align with current clinical evidence. This rigorous parameterization, together with multidisciplinary expert validation, enhances model validity and reliability. Additionally, sensitivity analyses and various scenario analyses provide actionable guidance for policymakers.

Our model also incorporates cardiovascular disease–related mortality, a leading cause of death in MASLD populations, addressing a gap in prior liver disease cost‐effectiveness analyses [13]. By including both liver‐specific and cardiovascular outcomes, this framework more accurately reflects real‐world patient trajectories than models focused solely on hepatic morbidity and mortality.

This study has limitations. First, we evaluated only a one‐time screening event, which yielded negligible QALY gains versus no screening. In addition, the 3 strategies produced similar total lifetime QALYs. This analysis framework was constrained to one-time screening, as Thailand—a middle-income country—has limited TE resources (approximately 120 devices nationwide), alongside shortages of trained personnel and infrastructure, rendering periodic screening currently infeasible. Nonetheless, one-time screening remains superior to no screening by enabling early fibrosis detection and raising awareness in high-risk populations. Further research should assess the long‐term benefits and costs of repeated or periodic screening. Our budget impact analysis included only screening costs; therefore, it may not fully capture the incremental budget required to implement screening compared with no screening. Overlap between target subgroups may have inflated the national budget impact. Because incidence data for individuals with concurrent MetS and obesity were unavailable, we approximated joint incidence by averaging published incidence rates for MetS and obesity. We then modeled a scenario restricted to those with both conditions. In that scenario, the annual budget impact decreased by approximately 50% compared with screening individuals with obesity using TE alone.

Due to limited empirical data on service capacity for liver fibrosis screening in Thailand, we could not appropriately model capacity-constrained scenarios. While we explored broad scenarios by varying various parameters, including screening uptake rates, future research incorporating explicit capacity and rollout constraints—as well as budget impact estimates using a phased implementation approach—is recommended to provide more realistic implementation projections and manage high budget impact.

Second, although multiple noninvasive devices exist to screen liver fibrosis and steatosis, limited availability within Thailand’s healthcare system constrained local data for a comprehensive economic evaluation. Consequently, our study focused on TE as the primary modality. While this choice reflects real‐world feasibility, it may limit generalizability to settings where alternative screening tools are more widely accessible. In addition, discrepancies remain regarding the optimal liver stiffness cutoff threshold for detecting significant fibrosis. Our analysis adopted a cutoff of ≥ 7.0 kPa based on evidence from previously published literature in Thailand [20] and globally [3,18,19], as well as expert consultation. The adoption of alternative cutoffs may alter the cost-effectiveness and budget impact of screening strategies. These results should therefore be interpreted with caution.

Third, our analyses relied primarily on cost data from the Siriraj Hospital database, which may limit generalizability. Siriraj Hospital is Thailand’s largest university hospital, with over 3 million outpatient visits and 80,000 inpatient admissions annually [36]. It routinely uses TE for liver fibrosis screening. In Thailand, MASLD care is typically provided by gastroenterologists in tertiary or university hospitals. Accordingly, treatment costs are expected to be similar across these centers, making the Siriraj database a relevant and practical source. Nonetheless, differences in resources and patient mix across levels of care should be considered when interpreting our results. Although the primary cost cohort included many individuals and yielded reasonable inputs, the model included numerous health states. Subgroup cost analyses reduced the number per health state, which may introduce greater uncertainty. Because direct extraction of cost data was not feasible and cost-to-charge ratios from university hospitals were limited, we converted charges to costs using a cost-to-charge ratio of 1.

Finally, our model did not include the incidence of HCC in noncirrhotic patients, reported at approximately 0.04%–0.6% per year in Asian populations with MASLD [53,58,59]. Given these relatively low rates compared with those in cirrhotic patients, this omission likely leads to a modest underestimation of the cost-effectiveness of fibrosis detection.

## Conclusions

In summary, screening for fibrosis stage ≥ 2 in individuals with MetS or obesity—particularly stepwise clinical scoring followed by TE—demonstrated favorable cost‐effectiveness. However, non-robustness in cost-effectiveness estimates and the substantial budget impact remain key considerations. Targeted strategies that prioritize high‐risk subgroups are essential to optimize resource allocation and improve population health outcomes.

## Supporting information

S1 File CHEERS 2022 checklists. (PDF)

S2 File A decision tree. (PDF)

S3 File Primary data analysis using data from Siriraj Hospital. (PDF)

S4 File Detailed calculation of treatment effectiveness. (PDF)

S5 File Cost analysis using primary data from the electronic database of Siriraj Hospital. (PDF)

S6 File Model validation process. (PDF)

S7 File Budget impact analysis: target population size estimation. (PDF)

S8 File Costs per true positive case detected and results of scenario analyses. (PDF)

S9 File Results of one-way sensitivity analyses: adherence rates and cost‑to‑charge ratios. (PDF)

S10 File Results of probabilistic sensitivity analyses. (PDF)

S11 File Results of the threshold analysis. (PDF)

S12 File Results of budget impact analyses. (PDF)
