# Supplementary material for: Cost-utility and budget impact analyses of significant fibrosis detection in individuals with metabolic syndrome or obesity in Thailand
Source: PLoS One. 2026 Mar 23;21(3):e0344985. doi: 10.1371/journal.pone.0344985 (PMC13008101; doi:10.1371/journal.pone.0344985)
Supplement: S2 File — (PDF) [file pone.0344985.s002.pdf]

S2 File. A decision tree

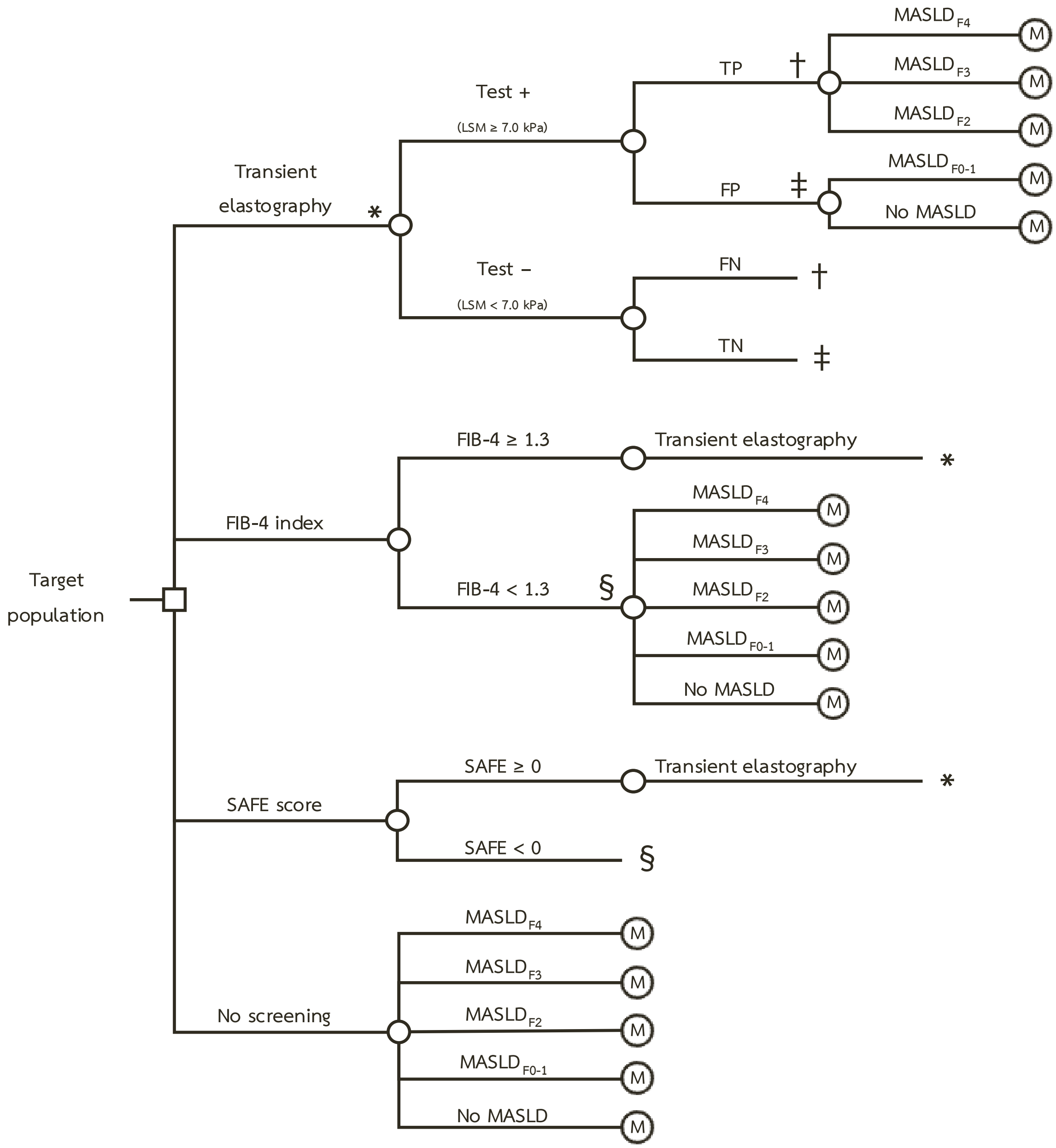

Figure S1 A decision tree

Abbreviations: F, fibrosis stage; FIB-4, fibrosis-4 index; FN, false negative; FP, false positive; LSM, liver stiffness measurement; M, Markov model; MASLD, metabolic dysfunction-associated steatotic liver disease; SAFE, steatosis-associated fibrosis estimator score; TE, transient elastography; TN, true negative; TP, true positive
