# Supplementary material for: Cost-utility and budget impact analyses of significant fibrosis detection in individuals with metabolic syndrome or obesity in Thailand
Source: PLoS One. 2026 Mar 23;21(3):e0344985. doi: 10.1371/journal.pone.0344985 (PMC13008101; doi:10.1371/journal.pone.0344985)
Supplement: S3 File — (PDF) [file pone.0344985.s003.pdf]

S3 File. Primary data analysis using data from Siriraj Hospital

The age-specific prevalence proportions of metabolic dysfunction-associated steatotic liver disease (MASLD) with significant fibrosis were calculated using data of 743 individuals with metabolic syndrome (MetS) and 799 individuals with obesity who underwent TE at Siriraj Hospital between 2018 and 2023 (**Table S1**). The significant fibrosis was defined in those who had liver stiffness measurement (LSM) of ≥ 7.0 kPa.

Table S1 Age-specific prevalence proportions of MASLD with significant fibrosis (LSM ≥ 7 kPa) from primary data analysis

Age group	Total N	LSM ≥ 7 kPa (n)	Prevalence (95% CI)
Metabolic Syndrome (N = 743)			
30–39.9 years	23	4	0.174 (0.050, 0.388)
40–49.9 years	65	7	0.108 (0.044, 0.209)
50–59.9 years	251	29	0.116 (0.079, 0.162)
60–69.9 years	263	30	0.114 (0.078, 0.159)
70–79.9 years	114	12	0.105 (0.056, 0.177)
≥ 80 years	27	11	0.407 (0.224, 0.612)
Obesity (N = 799)			
30–39.9 years	60	15	0.250 (0.147, 0.379)
40–49.9 years	97	22	0.227 (0.148, 0.323)
50–59.9 years	220	53	0.241 (0.186, 0.303)
60–69.9 years	288	104	0.361 (0.306, 0.420)
70–79.9 years	111	36	0.324 (0.239, 0.420)
≥ 80 years	23	12	0.522 (0.306, 0.732)

Abbreviations: CI, confidence interval; kPa, kilopascal; LSM, liver stiffness measurement; N, total screened cases (denominators); n, number of cases with MASLD and significant fibrosis (numerators)
