# Supplementary material for: Cost-utility and budget impact analyses of significant fibrosis detection in individuals with metabolic syndrome or obesity in Thailand
Source: PLoS One. 2026 Mar 23;21(3):e0344985. doi: 10.1371/journal.pone.0344985 (PMC13008101; doi:10.1371/journal.pone.0344985)
Supplement: S4 File — (PDF) [file pone.0344985.s004.pdf]

S4 File. Detailed calculation of treatment effectiveness

Weight-loss heterogeneity was explicitly modeled rather than averaged as a single weight-loss category. Individuals receiving the lifestyle intervention were stratified into four weight-loss groups based on the magnitude of weight loss achieved: (1) < 5%, (2) 5 – < 7%, (3) 7 – < 10%, and (4) ≥ 10%. The probability of being in each weight loss achievement category, and the corresponding probability of fibrosis regression, were derived from Vilar-Gomez et al. (2015). The weighted average proportion of patients experiencing fibrosis regression across these four categories was 0.204, which was the relative risk reduction of fibrosis progression, as summarized in **Table S2**.

Table S2 Relative risk reduction for fibrosis progression based on weight loss achievement from a lifestyle modification program

% Weight loss achievement	Probabilities of weight loss achievement	Probability of fibrosis regression (95% CI)
< 5%	64.60%	0.161 (0.113 - 0.219)
5 - < 7%	14.16%	0.176 (0.068 - 0.345)
7 - < 10%	7.08%	0.160 (0.045 - 0.361)
≥ 10%	14.16%	0.448 (0.264 - 0.643)
Weighted average probability of fibrosis regression (Relative risk reduction of fibrosis progression[a])		0.204 (0.153 - 0.254)

[a] Assuming that patients who did not receive lifestyle modification have no benefit on liver fibrosis regression and follow the natural history of disease progression, the risk of fibrosis progression under usual care equals 1.

We did not modify the transition probabilities for fibrosis regression. The effect of lifestyle intervention was conservatively applied only to the fibrosis progression pathways, in order to avoid overestimating clinical benefits of the intervention. Patients who did not receive lifestyle modification were assumed to follow the natural history of fibrosis progression, with no additional benefit in terms of fibrosis regression.
