# Supplementary material for: Cost-utility and budget impact analyses of significant fibrosis detection in individuals with metabolic syndrome or obesity in Thailand
Source: PLoS One. 2026 Mar 23;21(3):e0344985. doi: 10.1371/journal.pone.0344985 (PMC13008101; doi:10.1371/journal.pone.0344985)
Supplement: S5 File — (PDF) [file pone.0344985.s005.pdf]

S5 File. Cost analysis using primary data from the electronic database of Siriraj Hospital

Primary data analysis from the electronic database of Siriraj Hospital of 780 patients with MetS or obesity who underwent TE between January 1, 2018 and October 31, 2023, and 91 patients with $MASLD_{F4}$ (**Table S3**).

Table S3 Cost analysis results based on primary data from the Siriraj Hospital electronic database

**Direct medical Cost**	**Total number of visits**	**Visit per year**	**Median cost, THB/visit (USD/visit)**	**SE, THB/visit (USD/visit)**	**P5, THB/visit (USD/visit)**	**P95, THB/visit (USD/visit)**	**Total treatment cost, THB/year (USD/year)**
Metabolic syndrome							
Outpatient department visit							
MetS without MASLD	138	0.76	1,634.50 (47.19)	41,311.06 (1,192.60)	363.75 (10.50)	18,737.19 (540.92)	1,238.18 (35.74)
MetS + $MASLD_{F0-F3}$	1,259	2.75	2,727.24 (78.73)	226.16 (6.53)	743.28 (21.46)	19,419.25 (560.61)	7,504.10 (216.63)
MetS + $MASLD_{F4}$	316	9.01	2,231.91 (64.43)	963.93 (27.83)	1,289.89 (37.24)	31,011.19 (895.26)	20,103.40 (580.36)
Inpatient department visits							
MetS without MASLD	2	0.01	111,897.38[c] (3,230.35)	52,004.79 (1,501.32)	45,706.09 (1,319.48)	178,088.66 (5,141.22)	1,241.34 (35.84)
MetS + $MASLD_{F0-F3}$	1	0.01	20,225.50[c] (583.89)	NA	NA	NA	112.19 (3.24)
MetS + $MASLD_{F4}$[b]	68	0.31	51,010.51 (1,472.62)	1,125.35 (32.49)	6,039.55 (174.35)	225,096.95 (6,498.29)	15,999.44 (461.89)

Direct medical Cost	Total number of visits	Visit per year	Median cost, THB/visit (USD/visit)	SE, THB/visit (USD/visit)	P5, THB/visit (USD/visit)	P95, THB/visit (USD/visit)	Total treatment cost, THB/year (USD/year)
Obesity							
Outpatient department visit							
Obesity without MASLD[a]	NA	0	0	0	0	0	0
Obesity + $MASLD_{F0-F3}$	9,084	5.38	2,445.00 (70.58)	110.54 (3.19)	597.50 (17.25)	13,309.66 (384.23)	13,166.12 (380.09)
Obesity + $MASLD_{F4}$	1,703	6.00	3,338.84 (96.39)	388.07 (11.20)	1,135.13 (32.77)	27,848.32 (803.95)	20,017.62 (577.89)
Inpatient department visits							
Obesity without MASLD[a]	NA	0	0	0	0	0	0
Obesity + $MASLD_{F0-F3}$	6	0.02	5,405.50 (156.05)	33,742.66 (974.11)	2,905.19 (83.87)	377,532.94 (10,898.95)	89.56 (2.59)
Obesity + $MASLD_{F4}$ [b]	68	0.31	51,010.51 (1,472.62)	1,125.35 (32.49)	6,039.55 (174.35)	225,096.95 (6,498.29)	15,999.44 (461.89)

[a] Assumption

[b] Assumed equal for individuals with metabolic syndrome and those with obesity

[c] The median IPD cost for MetS without MASLD and for MetS + $MASLD_{F0-F3}$ were calculated based on a small number of visits. This limited data might introduce high uncertainty when applying these costs in the model. The frequency of IPD visit is relatively low due to the natural characteristic of the disease, with only one patient out of 780 in the cohort having IPD visits. This particular patient also had multiple comorbidities leading to a prolonged hospital stay and high IPD costs. However, when adjusting for visit frequency and

calculating the total cost per year, the overall value was minimal and unlikely to significantly affect the cost-effectiveness results. Furthermore, extreme value testing was performed by assuming the IPD cost for MetS without MASLD was zero, which did not alter the cost-effectiveness outcomes.

Abbreviations: CC, compensated cirrhosis; F, fibrosis stage; MASLD, metabolic dysfunction-associated steatotic liver disease; NA, not applicable; P, percentile; SE, standard error; THB, Thai baht; USD, United States dollars

Using **Table S3** and direct non-medical costs (i.e., food and travel costs) from the Standard Cost Lists for Health Technology Assessment in Thailand (**Table 1**), annual treatment costs for each relevant health state were calculated as follows (all values were originally computed in Excel and are presented rounded to two decimal places):

Treatment costs per year

= (frequency of OPD visit per year * median cost of OPD visit per year)
+ frequency of IPD visit per year * median cost of IPD visit per year
+ (frequency of OPD visit per year + frequency of IPD visit per year) * (food + travel costs per visit)

Treatment costs per year for MetS

= (0.76 * 1,634.50) + (0.01 * 111,897.38) + (0.76 + 0.01) * (66.18 + 179.67) = 2,668.49

Treatment costs per year for MetS with $MASLD_{F0\text{-}F3}$

= (2.75 * 2,727.24) + (0.01 * 20,225.50) + (2.75 + 0.01) * (66.18 + 179.67) = 8,294.13

Treatment costs per year for Obesity with $MASLD_{F0\text{-}F3}$

= (5.38 * 2,445.00) + (0.02 * 5,405.50) + (5.38 + 0.02) * (66.18 + 179.67) = 14,583.67

Treatment costs per year for MetS with $MASLD_{F4}$

= (9.01 * 2,231.91) + (0.31 * 51,010.51) + (9.01 + 0.31) * (66.18 + 179.67) = 38,394.46

Treatment costs per year for Obesity with $MASLD_{F4}$

= (6.00 * 3,338.84) + (0.31 * 51,010.51) + (6.00 + 0.31) * (66.18 + 179.67) = 37,568.1
