# Supplementary figures and images for: Cost-utility and budget impact analyses of significant fibrosis detection in individuals with metabolic syndrome or obesity in Thailand
Source: PLoS One. 2026 Mar 23;21(3):e0344985. doi: 10.1371/journal.pone.0344985 (PMC13008101; doi:10.1371/journal.pone.0344985)

## S6 File. Model validation process

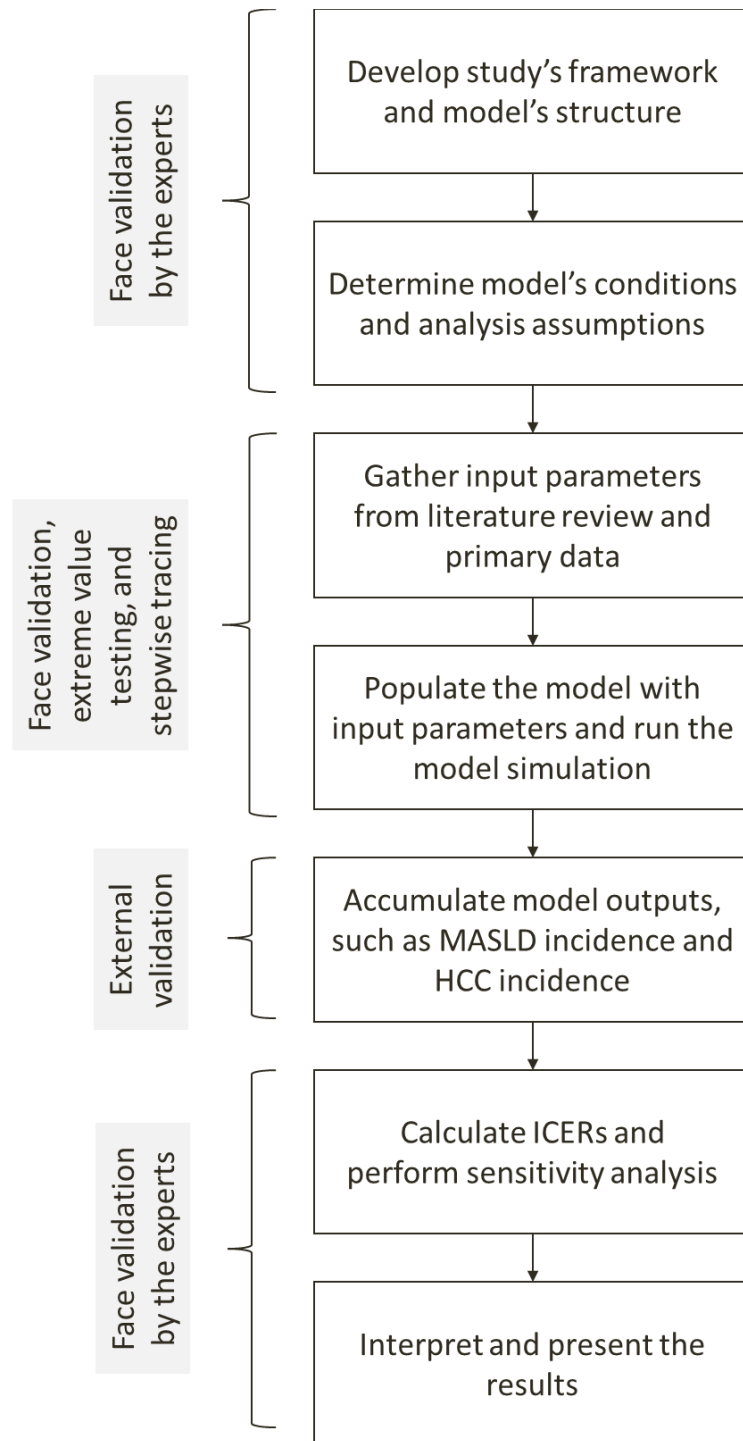

**Figure S2** Model validation process

Supplement: S6 File — (PDF) [file pone.0344985.s006.pdf]
