# Supplementary material for: Cost-utility and budget impact analyses of significant fibrosis detection in individuals with metabolic syndrome or obesity in Thailand
Source: PLoS One. 2026 Mar 23;21(3):e0344985. doi: 10.1371/journal.pone.0344985 (PMC13008101; doi:10.1371/journal.pone.0344985)
Supplement: S7 File — (PDF) [file pone.0344985.s007.pdf]

S7 File. Budget impact analysis: target population size estimation

The 5-year BIA was conducted from the payer perspective, focusing exclusively on the additional budget needed for screening fibrosis stage ≥ 2 (i.e., FIB-4+TE, SAFE+TE, or TE alone) among individuals with MetS, obesity or both conditions. Treatment and direct non-medical costs were excluded.

The target population consisted of individuals aged 50–79 years with MetS, obesity, or both conditions. For year 1, the prevalent populations were estimated by multiplying prevalence proportions by the total Thai population aged 50–79 years (21,348,831 individuals) [47]. For years 2 to 5, incident populations were estimated using age-specific incidence rates of the diseases multiplied by the populations aged 46 to 49 years (approximately 1,000,000 individuals per year) (**Table S4**).

The number of patients evaluated using scoring systems was calculated by applying screening rates of 90% (range, 50%–100%) for scoring systems and 80% (range, 30%–100%) for TE alone to the target populations. Subsequently, patients testing positive (FIB-4 ≥ 1.3 or SAFE ≥ 0) were estimated by multiplying age-specific test positivity proportions, obtained from primary data at Siriraj Hospital (**Table S5**), by the number evaluated via scoring systems. Our model assumed that 90% (range, 65%–100%) of score-positive individuals would undergo TE follow-up.

Budget impact was calculated by multiplying the number of individuals screened and/or undergoing TE by the corresponding unit costs (**Table 1**), without discounting over the 5-year horizon.

Table S4 Target population for budget impact analysis

Age (years)	Number of Thai populations	Patients with MetS	Patients with obesity	Patients with MetS and obesity
46	1,046,481	82,274[a]	9,667[a]	45,971[a]
47	1,033,245	80,932[a]	9,510[a]	45,221[a]
48	1,011,655	82,660[a]	9,713[a]	46,186[a]
49	1,028,431	83,718[a]	9,837[a]	46,778[a]
50	1,005,887	181,060	335,937	152,851
51	1,034,917	186,285	345,632	157,263
52	1,048,633	188,754	350,213	159,347
53	1,024,054	184,330	342,004	155,612
54	1,004,763	180,857	335,561	152,680
55	1,037,715	186,789	346,566	157,688
56	986,678	177,602	329,521	149,932

Age (years)	Number of Thai populations	Patients with MetS	Patients with obesity	Patients with MetS and obesity
57	962,437	173,239	321,426	146,249
58	970,565	174,702	324,140	147,484
59	962,074	173,173	321,304	146,194
60	898,136	161,664	299,951	136,478
61	870,877	156,758	290,847	132,336
62	827,157	148,888	276,246	125,692
63	821,545	147,878	274,372	124,839
64	780,182	140,433	260,558	118,554
65	713,405	128,413	238,256	108,407
66	682,631	122,874	227,979	103,730
67	623,156	112,168	208,116	94,693
68	576,621	103,792	192,574	87,621
69	584,686	105,243	195,268	88,847
70	536,360	96,545	179,128	81,503
71	518,958	93,412	173,317	78,859
72	481,509	86,672	160,810	73,168
73	452,275	81,410	151,047	68,726
74	430,799	77,544	143,874	65,463
75	375,907	67,663	125,542	57,122
76	333,581	60,045	111,406	50,690
77	299,120	53,842	99,897	45,453
78	261,149	47,007	87,216	39,683
79	243,054	43,750	81,173	36,934
**Total populations or patients aged 50-79**	**21,348,831**	**3,842,790**[b]	**7,129,882**[b]	**3,244,096**[b]

[a] Incident populations were estimated using age-specific incidence data of the diseases multiplied by the populations aged 46 to 49 years. Incidence rates were 8.0% for MetS [48], 0.9% for obesity [51], and 4.5% for both conditions combined [48, 51].

[b] Prevalent populations were estimated by multiplying prevalence proportions by the total Thai population aged 50–79 years. The prevalence proportions were 18.0% for MetS [48], 33.4% for obesity [49], and 15.2% for both conditions [49, 50].

Abbreviations: MetS, metabolic syndrome

Table S5 Age-specific proportion of patients with test positive

Age group (years)	Total *n* for FIB-4	FIB-4 ≥ 1.3	Proportion of individuals with FIB-4 ≥ 1.3 (95% CI)	Total *n* for SAFE	SAFE ≥ 0	Proportion of individuals with SAFE ≥ 0 (95% CI)
Metabolic syndrome						
40–59.9	307	44	0.143 (0.106, 0.188)	263	148	0.563 (0.500, 0.624)
60–79.9	370	231	0.624 (0.573, 0.674)	334	290	0.868 (0.827, 0.903)
Obesity						
40–59.9	317	77	0.243 (0.197, 0.294)	317	206	0.650 (0.595, 0.702)
60–79.9	399	260	0.652 (0.603, 0.698)	399	372	0.932 (0.903, 0.955)
Metabolic syndrome and obesity						
40–59.9	213	29	0.136 (0.093, 0.190)	183	65	0.355 (0.286, 0.429)
60–79.9	208	120	0.577 (0.507, 0.645)	184	174	0.946 (0.902, 0.974)

Abbreviations: CI, confidence interval; FIB-4, fibrosis-4 index; *n*, number; SAFE, steatosis-associated fibrosis estimator score

References

47. The Bureau of Registration Administration (BORA). Official statistics registration systems, 2012-2023 [Internet]. 2019 [cited March 20, 2024]. Available from: https://stat.bora.dopa.go.th/stat/statnew/statMONTH/statmonth/#/displayData.
48. Sakboonyarat B, Rangsin R, Mittleman MA. Incidence and risk factors of metabolic syndrome among Royal Thai Army personnel. Scientific reports. 2022;12(1):15692. Epub 2022/09/21. doi: 10.1038/s41598-022-19024-8. PubMed PMID: 36127360; PubMed Central PMCID: PMCPMC9489720.
49. Feng X, Sarma H, Seubsman SA, Sleigh A, Kelly M. Impact of age and gender differences in the prevalence and patterns of multimorbidity in the Thai Cohort Study. Int Health. 2024;16(4):454-62. Epub 2024/03/23 20:48. doi: 10.1093/inthealth/ihae018. PubMed PMID: 38520373; PubMed Central PMCID: PMCPMC11218883.
50. Kingkaew N, Howteerakul N, Tipayamongkholgul M, Suwannapong N, Rawdaree P. Prevalence and risk factors of metabolic syndrome among adults in a northeastern province of Thailand. Suranaree Journal of Science & Technology. 2019;26(4).

51. Liampeng S, Wongkliawrian N, Junlawakkananon S, Prapaso A, Panichnantho N, Kiengsiri S, et al. Effect of the weight-loss program using daily self-weighing combined with personalized counseling led by village health volunteers in adults with obesity in a rural community, Thailand: a randomized controlled trial. BMC Prim Care.
