# Supplementary material for: Cost-utility and budget impact analyses of significant fibrosis detection in individuals with metabolic syndrome or obesity in Thailand
Source: PLoS One. 2026 Mar 23;21(3):e0344985. doi: 10.1371/journal.pone.0344985 (PMC13008101; doi:10.1371/journal.pone.0344985)
Supplement: S8 File — (PDF) [file pone.0344985.s008.pdf]

S8 File. Costs per true positive case detected and results of scenario analyses

Table S6 Costs per true positive case detected

Screening strategy	% True positive among individuals with test positive[a]	% True positive among screened individuals[a]	Total costs of screeings[a, b], THB (USD)	Cost per 1 true positive case detected, THB (USD)
Metabolic syndrome				
FIB-4+TE	39.9%	5.8%	868.91 (25.08)	15,003.47 (433.13)
SAFE+TE	30.9%	8.0%	1,413.20 (40.80)	17,771.56 (513.04)
TE alone	27.9%	9.3%	1,600.00 (46.19)	17,288.73 (499.11)
Obesity				
FIB-4+TE	61.5%	12.1%	931.88 (26.90)	7,677.63 (221.64)
SAFE+TE	51.9%	16.6%	1,458.70 (42.11)	8,766.24 (253.07)
TE alone	48.5%	19.3%	1,600.00 (46.19)	8,291.52 (239.37)

[a] Calculated at screening rates of 90% for initial FIB-4 index and SAFE score screening with 90% of those with FIB-4 ≥ 1.3 and SAFE ≥ 0 proceeded to TE, and 80% for the TE alone screening strategy.

[b] Only costs of screening tests were considered: FIB-4 index = 271.0 THB (7.8 USD), SAFE score = 355.4 THB (10.3 USD), and TE = 2,000.0 THB (57.7 USD)

Abbreviations: FIB-4, fibrosis-4 index; SAFE, steatosis-associated fibrosis estimator score; TE, transient elastography; THB, Thai baht; USD, United State dollars

Table S7 Outcomes of scenario analyses across screening ages ranging from 30 to 80 years.

Outcome	Life expectancy (years)	Total lifetime cost, THB	Total QALYs	ICER[a], THB per QALY gained
Metabolic syndrome				
No screening at				
30 years	39.31	148,172.57	18.79	
40 years	33.24	130,183.61	17.27	
50 years[b]	26.81	109,897.26	15.22	
60 years	20.32	84,457.73	12.66	
70 years	14.19	60,405.04	9.64	
80 years	8.82	43,933.05	6.42	
Screening by FIB-4+TE at				
30 years	39.36	149,196.11	18.81	60,924.86
40 years	33.26	131,092.85	17.28	94,334.94
50 years[b]	26.83	110,748.62	15.23	104,587.67
60 years	20.33	85,105.57	12.66	182,208.72
70 years	14.19	60,989.96	9.64	338,074.62
80 years	8.82	44,584.36	6.43	249,818.79
Screening by SAFE+TE at				
30 years	39.38	149,813.65	18.82	71,164.94
40 years	33.28	131,672.57	17.29	108,546.06
50 years[b]	26.83	111,200.09	15.23	128,274.36
60 years	20.33	85,438.00	12.66	220,300.92
70 years	14.19	61,261.20	9.64	396,357.19
80 years	8.83	44,821.22	6.43	288,221.15
Screening by TE alone at				
30 years	39.37	150,835.83	18.81	135,633.63
40 years	33.27	132,672.89	17.28	232,978.33
50 years[b]	26.83	112,183.37	15.23	255,221.17
60 years	20.33	86,438.45	12.66	492,674.31
70 years	14.19	62,248.61	9.64	925,926.77
80 years	8.83	45,641.58	6.43	570,311.72

Outcome	Life expectancy (years)	Total lifetime cost, THB	Total QALYs	ICER[a], THB per QALY gained
Obesity				
No screening at				
30 years	42.47	220,108.65	20.67	
40 years	36.02	197,763.85	19.08	
50 years[b]	29.05	168,147.37	16.84	
60 years	21.67	130,931.74	13.73	
70 years	15.29	93,332.38	10.55	
80 years	9.87	67,756.23	7.25	
Screening by FIB-4+TE at				
30 years	42.52	220,797.18	20.69	38,310.43
40 years	36.05	198,450.10	19.09	46,899.42
50 years[b]	29.07	168,700.92	16.85	46,413.47
60 years	21.69	131,423.89	13.74	39,157.94
70 years	15.31	93,830.13	10.56	69,396.65
80 years	9.88	68,340.62	7.26	90,062.10
Screening by SAFE+TE at				
30 years	42.54	221,177.69	20.70	43,144.97
40 years	36.07	198,831.35	19.10	52,766.63
50 years[b]	29.08	169,013.48	16.86	51,743.86
60 years	21.70	131,687.12	13.75	41,761.28
70 years	15.31	94,069.78	10.56	71,108.63
80 years	9.89	68,586.97	7.26	90,423.49
Screening by TE alone at				
30 years	42.55	221,704.99	20.70	53,428.83
40 years	36.08	199,377.54	19.10	65,989.20
50 years[b]	29.09	169,492.42	16.86	65,728.93
60 years	21.71	132,036.11	13.75	52,184.99
70 years	15.31	94,594.77	10.56	103,374.22
80 years	9.89	69,164.54	7.26	134,224.20

[a] Compared to no screening. ICERs shown in green represent cost-effective strategies, with values at or below the WTP threshold of 160,000 THB per QALY gained, while ICERs in red indicate strategies that are not cost-effective, exceeding the WTP threshold.

[b] Based-case analysis

Abbreviations: FIB-4, fibrosis-4 index; ICER, incremental cost-effectiveness ratio; QALY, quality-adjusted life-year; SAFE, steatosis-associated fibrosis estimator score; TE, transient elastography; THB, Thai baht; USD, United State dollars; WTP, willingness-to-pay
