# Supplementary material for: Cost-utility and budget impact analyses of significant fibrosis detection in individuals with metabolic syndrome or obesity in Thailand
Source: PLoS One. 2026 Mar 23;21(3):e0344985. doi: 10.1371/journal.pone.0344985 (PMC13008101; doi:10.1371/journal.pone.0344985)
Supplement: S9 File — (PDF) [file pone.0344985.s009.pdf]

S9 File. Results of one-way sensitivity analyses: adherence rates and cost-to-charge ratios

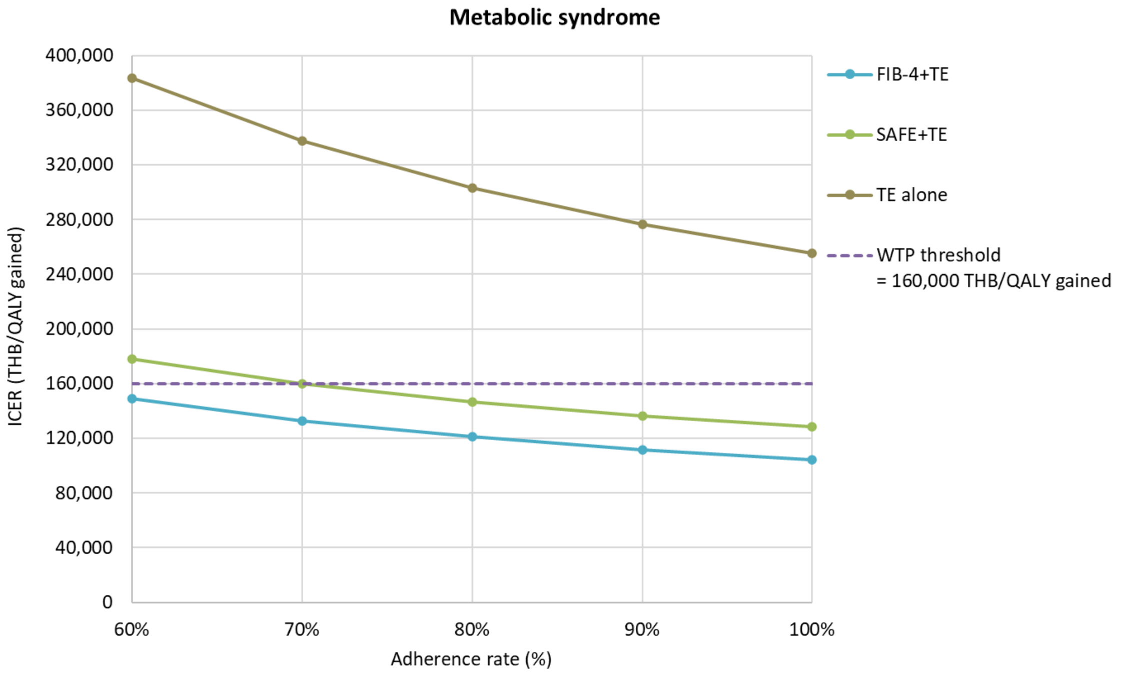

(a)

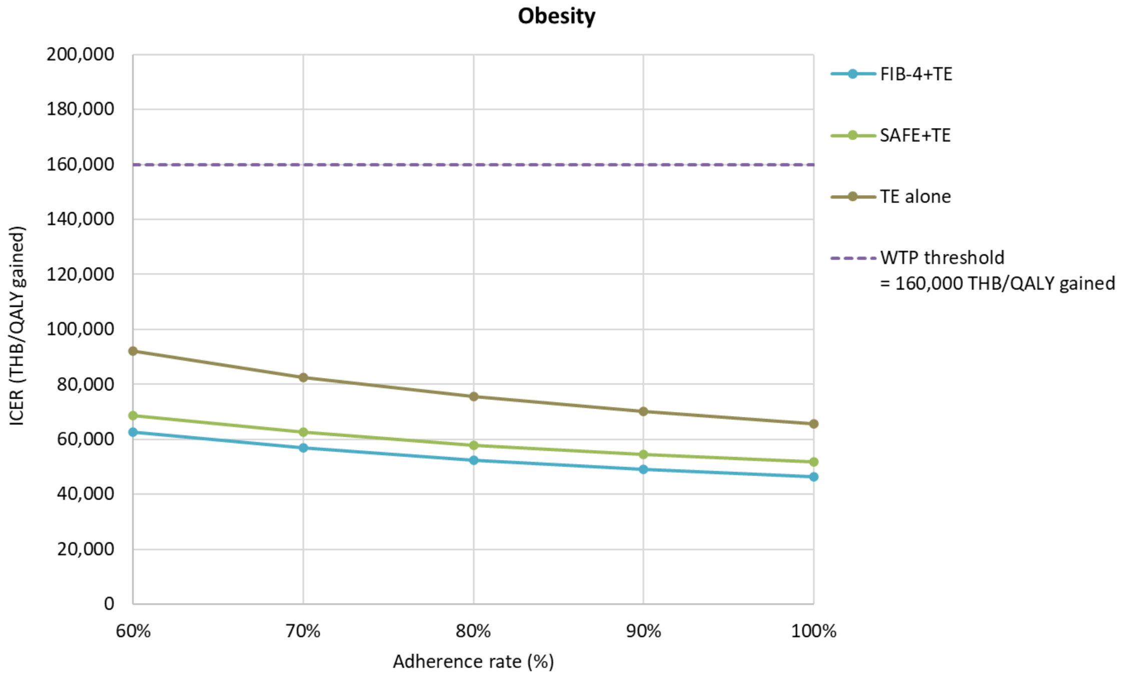

(b)

Figure S3 Impact of lifestyle intervention adherence rates (ranging from 60% to 100%) on the ICER of each screening strategy, compared to no screening, in (a) individuals with metabolic syndrome and (b) individuals with obesity

Abbreviations: FIB-4, fibrosis-4 index; ICER; incremental cost-effectiveness ratio; QALY, quality-adjusted life-year; SAFE, steatosis-associated fibrosis estimator score; TE, transient elastography; THB, Thai baht; WTP, willingness-to-pay

Table S8 Unit costs and ICERs across alternative cost-to-charge ratios

	**RCC = 1 (base-case analysis)**	**RCC = 0.6**	**RCC = 1.2**
TE, THB (USD)	2,000.0 (57.7)	1,200.0 (34.6)	2,400.0 (69.3)
Metabolic syndrome			
Treatment costs per year, THB (USD)			
MetS	2,668.5 (77.0)	1,601.1 (46.2)	3,202.2 (92.4)
MetS with $MASLD_{F0-F3}$	8,294.1 (239.4)	4,976.5 (143.7)	9,953.0 (287.3)
MetS with $MASLD_{F4}$	38,394.5 (1,108.4)	23,036.7 (665.0)	46,073.4 (1,330.1)
ICERs (compared to no screening), THB (USD) per QALY gained			
FIB-4+TE	104,587.67 (3,019.33)	114,328.05 (3,300.52)	99,717.48 (2,878.73)
SAFE+TE	128,274.36 (3,703.13)	134,129.10 (3,872.15)	125,346.99 (3,618.62)
TE alone	255,221.17 (7,367.94)	204,863.96 (5,914.19)	280,399.77 (8,094.82)
Obesity			
Treatment costs per year, THB (USD)			
Obesity	0	0	0
Obesity with $MASLD_{F0-F3}$	14,583.7 (421.0)	8,750.2 (252.6)	17,500.4 (505.2)
Obesity with $MASLD_{F4}$	37,568.2 (1,084.6)	22,540.9 (650.7)	45,081.8 (1,301.5)
ICERs (compared to no screening), THB (USD) per QALY gained			
FIB-4+TE	46,413.47 (1,339.90)	42,249.44 (1,219.69)	48,495.48 (1,400.01)
SAFE+TE	51,743.86 (1,493.79)	47,324.80 (1,366.21)	53,953.40 (1,557.57)
TE alone	65,728.93 (1,897.52)	50,474.44 (1,457.14)	73,356.17 (2,117.71)

Abbreviations: F, fibrosis stage; FIB-4, fibrosis-4 index; ICERs; incremental cost-effectiveness ratios; MASLD; metabolic dysfunction-associated steatotic liver disease; MetS, metabolic syndrome; QALY, quality-adjusted life-year; RCC, ratio of cost to charge; SAFE, steatosis-associated fibrosis estimator score; TE, transient elastography; THB, Thai baht; USD, Unite States dollars
