# Supplementary material for: Cost-utility and budget impact analyses of significant fibrosis detection in individuals with metabolic syndrome or obesity in Thailand
Source: PLoS One. 2026 Mar 23;21(3):e0344985. doi: 10.1371/journal.pone.0344985 (PMC13008101; doi:10.1371/journal.pone.0344985)
Supplement: S10 File — (PDF) [file pone.0344985.s010.pdf]

S10 File. Results of probabilistic sensitivity analyses

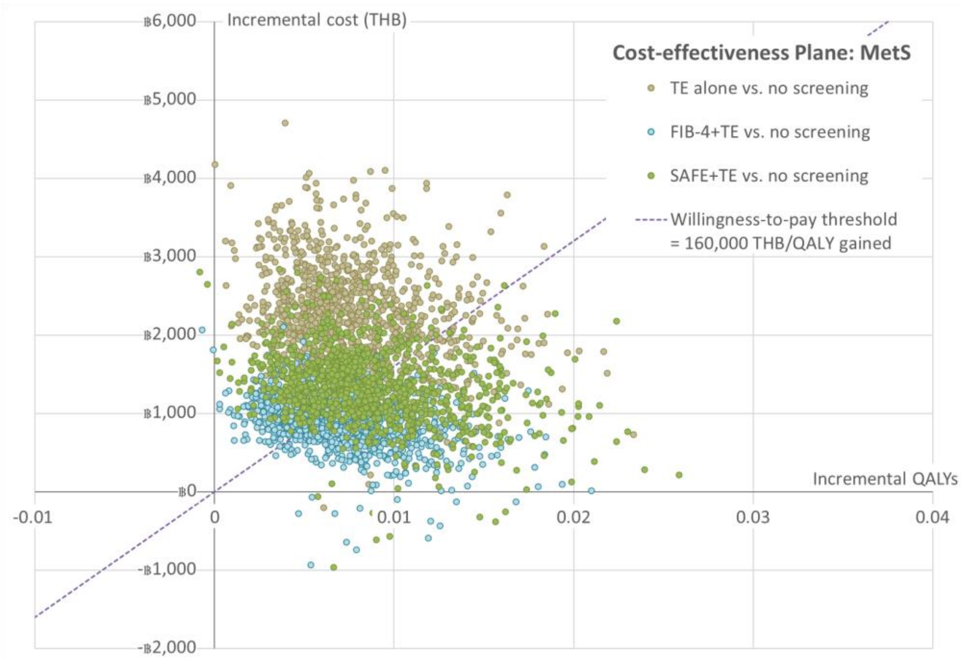

(a)

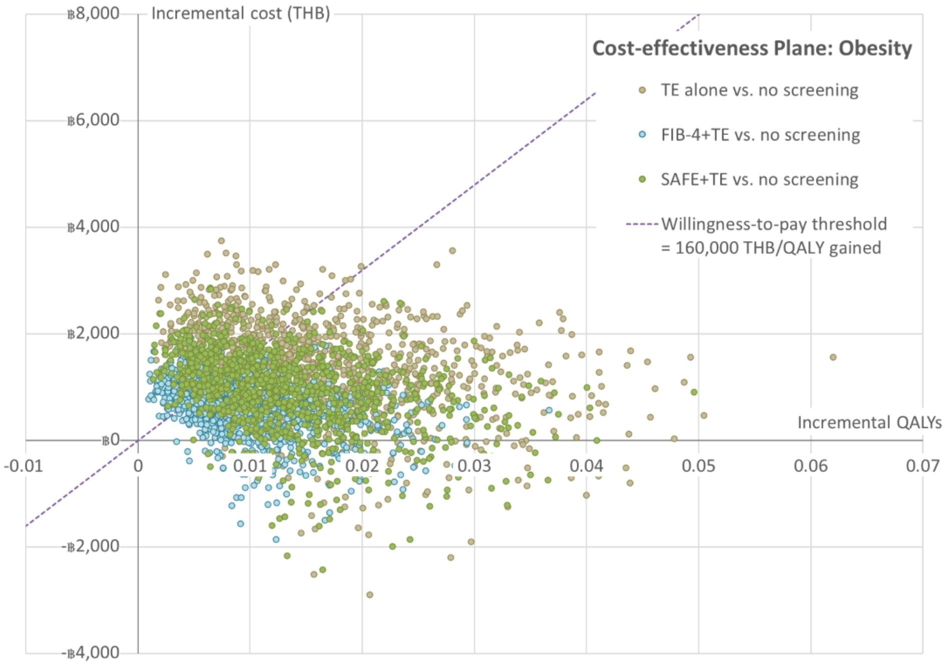

(b)

Figure S4 Cost-effectiveness planes of (a) patients with metabolic syndrome and (b) patients with obesity

Abbreviations: FIB-4, fibrosis-4 index; QALY, quality-adjusted life-year; MetS, metabolic syndrome; SAFE, steatosis-associated fibrosis estimator score; TE, transient elastography; THB, Thai baht
