# Supplementary material for: Cost-utility and budget impact analyses of significant fibrosis detection in individuals with metabolic syndrome or obesity in Thailand
Source: PLoS One. 2026 Mar 23;21(3):e0344985. doi: 10.1371/journal.pone.0344985 (PMC13008101; doi:10.1371/journal.pone.0344985)
Supplement: S11 File — (PDF) [file pone.0344985.s011.pdf]

S11 File. Results of the threshold analysis

Table S9 ICERs of TE alone compared with no screening, among individuals with metabolic syndrome, across a range of TE unit costs

% Reduction	Unit cost of TE, THB (USD)	ICERs[a], THB (USD) per QALY gained
0% (base-case analysis)	2,000.00 (57.74)	255,221.17 (7,367.94)
10%	1,800.00 (51.96)	237,725.20 (6,862.86)
20%	1,600.00 (46.19)	220,229.23 (6,357.77)
30%	1,400.00 (40.42)	202,733.27 (5,852.68)
40%	1,200.00 (34.64)	185,237.30 (5,347.59)
50%	1,000.00 (28.87)	167,741.33 (4,842.50)
54.4%	911.51 (26.31)	160,000.00 (4,619.02)
60%	800.00 (23.10)	150,245.36 (4,337.41)

[a] ICERs of TE alone compared to no screening, among individuals with metabolic syndrome. ICERs shown in green represent cost-effective strategies, with values at or below the willingness-to-pay threshold of 160,000 THB (4,619 USD) per QALY gained, while ICERs in red indicate strategies that are not cost-effective, exceeding the willingness-to-pay threshold.

Abbreviations: ICERs, cost-effectiveness ratios; QALY, quality-adjusted life-year; TE, transient elastography; THB, Thai baht; USD, United States dollars
