# Supplementary material for: Cost-utility and budget impact analyses of significant fibrosis detection in individuals with metabolic syndrome or obesity in Thailand
Source: PLoS One. 2026 Mar 23;21(3):e0344985. doi: 10.1371/journal.pone.0344985 (PMC13008101; doi:10.1371/journal.pone.0344985)
Supplement: S12 File — (PDF) [file pone.0344985.s012.pdf]

S12 File. Results of budget impact analyses

Table S10 Budget impact analysis for significant fibrosis screening in individuals with metabolic syndrome or obesity aged between 50 and 79 years

Parameter	Y1	Y2	Y3	Y4	Y5	Total
Total population	21,348,831					21,348,831
New population		1,028,431	1,011,655	1,033,245	1,046,481	4,119,812
Metabolic syndrome						
Patients with MetS	3,842,790	82,274	80,932	82,660	83,718	4,172,375
Patients evaluated with scoring system	3,458,511	74,047	72,839	74,394	75,347	3,755,137
FIB-4+TE						
Patients with FIB-4 ≥ 1.3	1,377,070	10,613	10,439	10,662	10,799	1,419,583
Patients undergo TE	1,239,363	9,551	9,396	9,596	9,719	1,277,625
Total BIA, THB (USD)	3,415,950,786 (98,614,606)	39,168,762 (1,130,758)	38,529,832 (1,112,312)	39,352,108 (1,136,051)	39,856,213 (1,150,603)	3,572,857,702 (103,144,330)
Average annual budget, THB (USD)						714,571,540 (20,628,866)
Minimum – maximum of average annual budget[a], million THB (million USD)						318.1–857.1 (9.2–247.4)
SAFE+TE						
Patients with SAFE ≥ 0	2,506,079	41,669	40,989	41,864	42,400	2,673,002
Patients undergo TE	2,255,472	37,502	36,890	37,678	38,160	2,405,702
Total BIA, THB (USD)	5,740,233,626 (165,714,003)	101,323,518 (2,925,094)	99,670,706 (2,877,380)	101,797,805 (2,938,787)	103,101,848 (2,976,433)	6,146,127,503 (177,431,696)
Average annual budget, THB (USD)						1,229,225,501 (35,486,339)
Minimum – maximum of average annual budget[a], million THB (million USD)						534.4–1,484.6 (15.4–428.6)
TE alone						
Patients undergo screening	3,074,232	65,820	64,746	66,128	66,975	3,337,900

Parameter	Y1	Y2	Y3	Y4	Y5	Total
Total BIA, THB (USD)	6,148,463,328 (177,499,129)	131,639,168 (3,800,273)	129,491,840 (3,738,282)	132,255,360 (3,818,062)	133,949,568 (3,866,971)	6,675,799,264 (192,722,716)
Average annual budget, THB (USD)						1,335,159,853 (38,544,543)
Minimum – maximum of average annual budget[a], million THB (million USD)						500.7–1,669.0 (14.5–48.2)
Obesity						
Patients with obesity	7,129,882	9,667	9,510	9,713	9,837	7,168,609
Patients evaluated with scoring system	6,416,894	8,701	8,559	8,741	8,853	6,451,748
FIB-4+TE						
Patients with FIB-4 ≥ 1.3	2,948,275	2,113	2,079	2,123	2,150	2,956,742
Patients undergo TE	2,653,448	1,902	1,871	1,911	1,935	2,661,067
Total BIA, THB (USD)	7,045,816,047 (203,404,679)	6,161,842 (177,885)	6,061,329 (174,984)	6,190,686 (178,718)	6,269,989 (181,007)	7,070,499,893 (204,117,274)
Average annual budget, THB (USD)						1,414,099,979 (40,823,455)
Minimum – maximum of average annual budget[a], million THB (million USD)						621.3–1,702.6 17.9–80.8
SAFE+TE						
Patients with SAFE ≥ 0	5,130,379	5,654	5,562	5,680	5,753	5,153,029
Patients undergo TE	4,617,341	5,089	5,006	5,112	5,178	4,637,726
Total BIA, THB (USD)	11,515,498,849 (332,439,328)	13,269,654 (383,080)	13,053,197 (376,831)	13,331,769 (384,873)	13,502,550 (389,803)	11,568,656,019 (333,973,915)
Average annual budget, THB (USD)						2,313,731,204 (66,794,783)
Minimum – maximum of average annual budget[a], million THB (million USD)						999.1–2,799.8 28.8–49.2
TE alone						
Patients undergo screening	5,703,906	7,734	7,608	7,770	7,870	5,734,887
Total BIA, THB (USD)	11,407,811,646 (329,330,521)	15,467,602 (446,532)	15,215,291 (439,248)	15,540,005 (448,622)	15,739,074 (454,369)	11,469,773,619 (331,119,292)

Parameter	Y1	Y2	Y3	Y4	Y5	Total
Average annual budget, THB (USD)						2,293,954,724 (66,223,858)
Minimum – maximum of average annual budget[a], million THB (million USD)						860.2–2,867.4 24.8–82.8
Metabolic syndrome and obesity						
Patients with MetS and obesity	3,244,096	45,971	45,221	46,186	46,778	3,428,252
Patients evaluated with scoring system	2,919,687	41,374	40,699	41,567	42,100	3,085,427
FIB-4+TE						
Patients with FIB-4 ≥ 1.3	1,079,356	5,633	5,541	5,659	5,732	1,101,921
Patients undergo TE	971,420	5,070	4,987	5,093	5,159	991,729
Total BIA, THB (USD)	2,734,048,617 (78,928,868)	21,351,409 (616,391)	21,003,120 (606,336)	21,451,353 (619,276)	21,726,148 (627,209)	2,819,580,646 (81,398,080)
Average annual budget, THB (USD)						563,916,129 (16,279,616)
Minimum – maximum of average annual budget[b], million THB (million USD)						550.3–577.4 (15.9–16.7)
SAFE+TE						
Patients with SAFE ≥ 0	1,950,442	14,696	14,456	14,764	14,954	2,009,311
Patients undergo TE	1,755,398	13,226	13,010	13,288	13,458	1,808,380
Total BIA, THB (USD)	4,548,566,785 (131,311,939)	41,157,955 (1,188,183)	40,486,577 (1,168,801)	41,350,612 (1,193,745)	41,880,318 (1,209,037)	4,713,442,247 (136,071,706)
Average annual budget, THB (USD)						942,688,449 (27,214,341)
Minimum – maximum of average annual budget[b], THB (USD)						916.4–968.7 (26.5–28.0)
TE alone						
Patients undergo screening	2,595,277	36,777	36,177	36,949	37,422	2,742,602
Total BIA, THB (USD)	5,190,554,299 (149,845,387)	73,553,385 (2,123,402)	72,353,566 (2,088,765)	73,897,682 (2,133,342)	74,844,321 (2,160,670)	5,485,203,253 (158,351,567)
Average annual budget, THB (USD)						1,097,040,651 (31,670,313)

Parameter	Y1	Y2	Y3	Y4	Y5	Total
Minimum – maximum of average annual budget[b], THB (USD)						1,050.0–1,143.6 (30.3–33.0)

[a] The minimum and maximum of average annual budget are calculated based on the minimum and maximum screening rate, respectively.

[b] The minimum and maximum of average annual budget are calculated based on the obesity incidence of 0.9% per year and the MetS incidence of 8.0% per year, respectively.

Abbreviations: BIA, budget impact analysis; FIB-4, fibrosis-4 index; MetS, metabolic syndrome; SAFE, steatosis-associated fibrosis estimator score; TE, transient elastography; THB, Thai baht; USD, United States dollars; Y, year
